# Learning Gains in Organic Chemistry from Full and Hybrid Specifications Grading

**DOI:** 10.1021/jacsau.6c00531

**Published:** 2026-09-03

**Authors:** Joseph D. Houck, Brandon J. Yik, Eric D. Nacsa

**Affiliations:** † Department of Chemistry, The Pennsylvania State University, University Park, Pennsylvania 16802, United States; ‡ Department of Chemistry, 1355 University of Georgia, Athens, Georgia 30602, United States

**Keywords:** specifications grading, alternative grading, chemical education

## Abstract

In recent years, alternative grading strategies to traditional points-based systems have gained popularity, particularly in STEM. Specifications grading is one type of alternative grading that focuses students on meeting course learning objectives rather than accumulating points, sets a threshold for satisfactory work, and often includes frequent, low-stakes assessments, feedback, and opportunities for reattempts or revision. However, the contribution of these features to student learning in authentic courses remains difficult to isolate. This study provides course-scale evidence that frequent, feedback-rich, reattemptable learning-target assessment is associated with higher standardized exam performance in large-enrollment Organic Chemistry I. Students in multiple sections that adopted this assessment structure, whether embedded in full specifications grading or in a hybrid points-based system, scored significantly higher on the same custom ACS Organic Chemistry Exam than students in a traditionally graded comparison section. Higher scores were observed in both same-instructor and cross-instructor comparisons. Improvements were concentrated among students with lower incoming preparation, as well as among women and students who did not identify exclusively as white. No corresponding losses were observed among students with higher incoming preparation, men, or students who identified exclusively as white. Overall, these findings suggest that frequent, feedback-rich, reattemptable learning-target assessments may be a useful mechanism within alternative grading systems for supporting student performance in organic chemistry.

## Graphical abstract

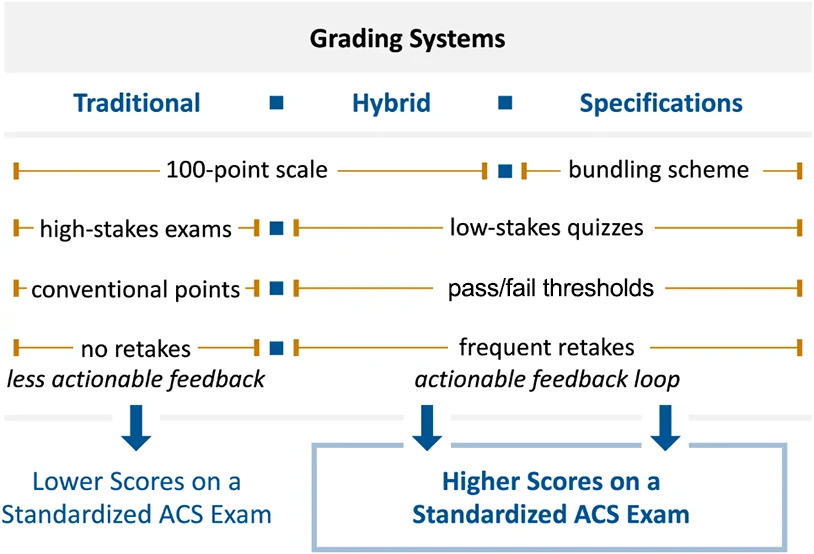

## Introduction

Specifications (“specs”) grading has emerged as a promising new pedagogical tool in the past decade since its establishment by Nilson in 2015.^1,2^ Designed as an alternative to traditional points-based grading, specs grading seeks to address many of the shortcomings associated with the traditional 100-point, A–F scales.^1,2^ Specs grading focuses on a handful of core principles, including: (1) clearly defined learning objectives aligned with graded assessments, (2) pass/fail evaluation of student work to incentivize mastery of key concepts, (3) cycles of feedback and revision for work that does not initially meet expectations, and (4) final course grades determined by tiers or “bundles” of assessments passed.^1,2^ These features are intended to make expectations more transparent, emphasize eventual achievement of learning outcomes, and shift students’ attention away from point accumulation toward demonstrated competency.^3^

Specs grading and related alternative grading approaches have been implemented across a wide range of instructional contexts and disciplines, as summarized in recent reviews.^4,5^ In chemistry, reports of specs grading have substantially increased in recent years.^6^ Implementation across the curriculum can be found in the literature, including both lectures and laboratories, in general chemistry,^7–14^ organic chemistry,^15–21^ analytical chemistry,^22,23^ inorganic chemistry,^24^ physical chemistry,^24,25^ biochemistry and chemical biology,^24,26,27^ and graduate-level coursework.^28^ Collectively, these reports suggest that specs grading is both adaptable and increasingly relevant across chemistry subdisciplines and course formats. Detailed discussions enumerate how adopting this grading scheme should support a range of improvements for student and instructor experiences (Figure 1a).^1,2^ Prior studies have reported instructor-perceived benefits, student perceptions of grading systems, and mixed evidence regarding equity-related outcomes.^6,10^ However, many claims about how specs grading affects student learning remain difficult to evaluate because course grades, student attitudes, and instructor reflections are often not directly comparable across grading systems.

**Figure 1:**
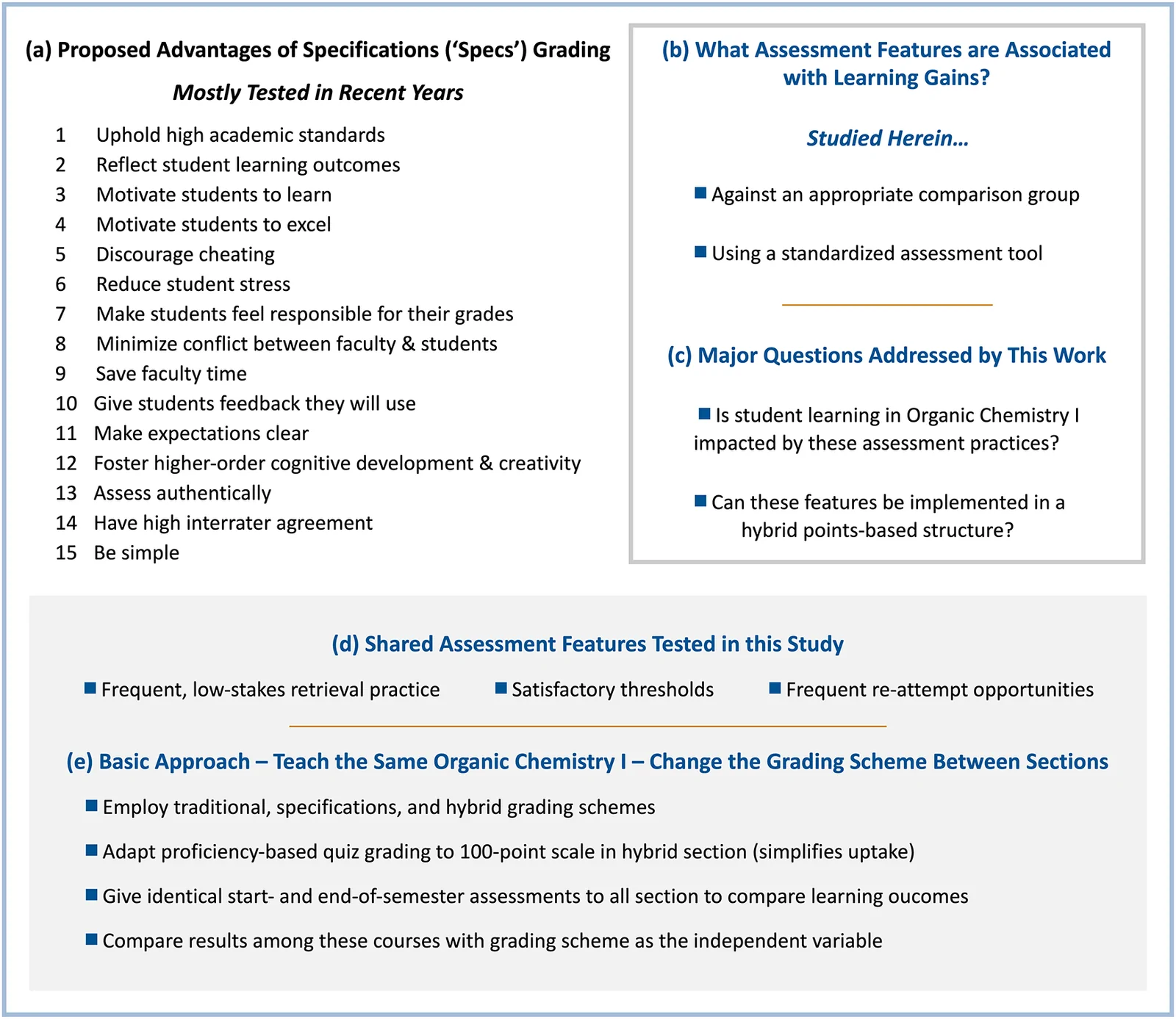
Proposed advantages of specifications grading and key questions addressed by this study.

Compared to these wide-ranging benefits centered on attitudes and experiences, less focus has been dedicated to whether specifications grading is associated with student learning gains, particularly when learning is measured using outcomes that can be directly compared across course sections. Several reports have explored academic outcomes in specifications-graded courses, but many have focused on final course grades or other course-specific measures that are difficult to compare with outcomes from traditionally graded courses or other comparison groups (Figure 1b).^7,8,16,21,26,28^ In fact, in their comprehensive review, Howitz et al. identified this as a major gap in the literature, arguing that future work beyond descriptive implementation studies should include systematic investigations of student academic performance: *“Many of the current publications on specifications grading are descriptive studies on individual designs and implementations. While additional descriptive publications will be valuable resources for practitioners seeking inspiration for their courses, especially from disciplines or types of courses that are not yet represented, enough evidence now exists to point to the fact that we need future research to include systematic studies on topics such as impacts on student academic performance···”* ^4^ We sought to address this gap by teaching multiple, otherwise-similar large-enrollment sections of Organic Chemistry I that differed mainly in their grading scheme (traditional grading, hybrid learning-target assessment, or full specifications grading), and using a custom ACS Organic Chemistry Exam taken by all sections to compare student learning outcomes.^29^

An additional challenge is that specs grading is not a single instructional intervention. It often combines several features that may influence student learning through distinct mechanisms. Many specs grading systems include frequent low-stakes assessment, repeated retrieval practice, feedback, and opportunities to act on feedback through revision or reattempts. These features are not unique to specs grading and are already supported by broader science learning literature. Retrieval practice has been shown to support long-term retention, feedback is a well-established contributor to learning, and frequent low-stakes testing has been associated with course performance.^30–32^ Thus, our question is whether a course-scale assessment structure built around them is associated with improved performance on an externally validated assessment in large-enrollment organic chemistry.

This distinction is important for interpreting the contribution of alternative grading studies. If similar learning gains are observed in both full specifications grading and a more limited hybrid implementation, then the shared assessment structure may be a more plausible contributor to those outcomes than the course grade determination alone. Conversely, if full specs grading produces stronger outcomes than a hybrid implementation, it would suggest that features beyond their shared assessment structures also matter. Comparing these approaches can therefore help clarify which aspects of alternative grading warrant further investigation, adoption, and refinement.

Another proposed outcome of specs grading is to improve equity among students.^33–36^ Nilson and others have argued that specs grading may support students who are less wellserved by traditional high-stakes assessment structures.^1,3,37^ In chemistry, recent work has begun to explore these questions, including studies examining student perceptions of grading systems,^11^ and the association between specs grading and opportunity gaps.^10^ These mixed results are important, but evidence remains limited when learning is measured using outcomes that can be compared across sections rather than course-specific grades or perceptions. We therefore also examined whether differences in performance on the same exam given to differently graded sections were similar among students grouped by incoming preparation, gender identity, and racial/ethnic background.

From an implementation perspective, we sought to determine whether a more limited specs implementation could produce outcomes similar to those observed in full specs grading (Figure 1c). Full specs grading can require substantial instructor effort and student buy-in.^2,6,17^ A hybrid model that retains a familiar points-based course-grade structure while adopting frequent learning-target assessments with feedback and reattempts may lower some barriers to adoption (Figure 1d).

In summary, we examine student learning outcomes in multiple large-enrollment Organic Chemistry I sections using a custom ACS Organic Chemistry Exam (Figure 1e). This study was guided by three research questions: (1) Are courses using frequent, low-stakes, learning-target-focused assessments with feedback and reattempts associated with higher performance on a custom ACS Organic Chemistry Exam than a traditionally graded course organized around high-stakes exams? (2) Are similar outcomes observed when this assessment structure is embedded in a hybrid points-based system versus a full specifications grading system? and (3) Are the observed associations similar across student subgroups? This work represents one of the first multisection studies in chemistry to evaluate assessment structures using a shared external assessment while also examining student subgroup outcomes.

## Methods

This study employed a multisection, quasi-experimental design to compare student learning outcomes under traditional, specifications, and hybrid grading systems in a large-enrollment Organic Chemistry I course. Four course sections taught by two instructors across two academic years were analyzed using a custom ACS Organic Chemistry Exam as a common end-of-semester assessment measure and an early semester quiz to control for differential preparation for the course. The detailed study design is summarized in Figure 2.

**Figure 2:**
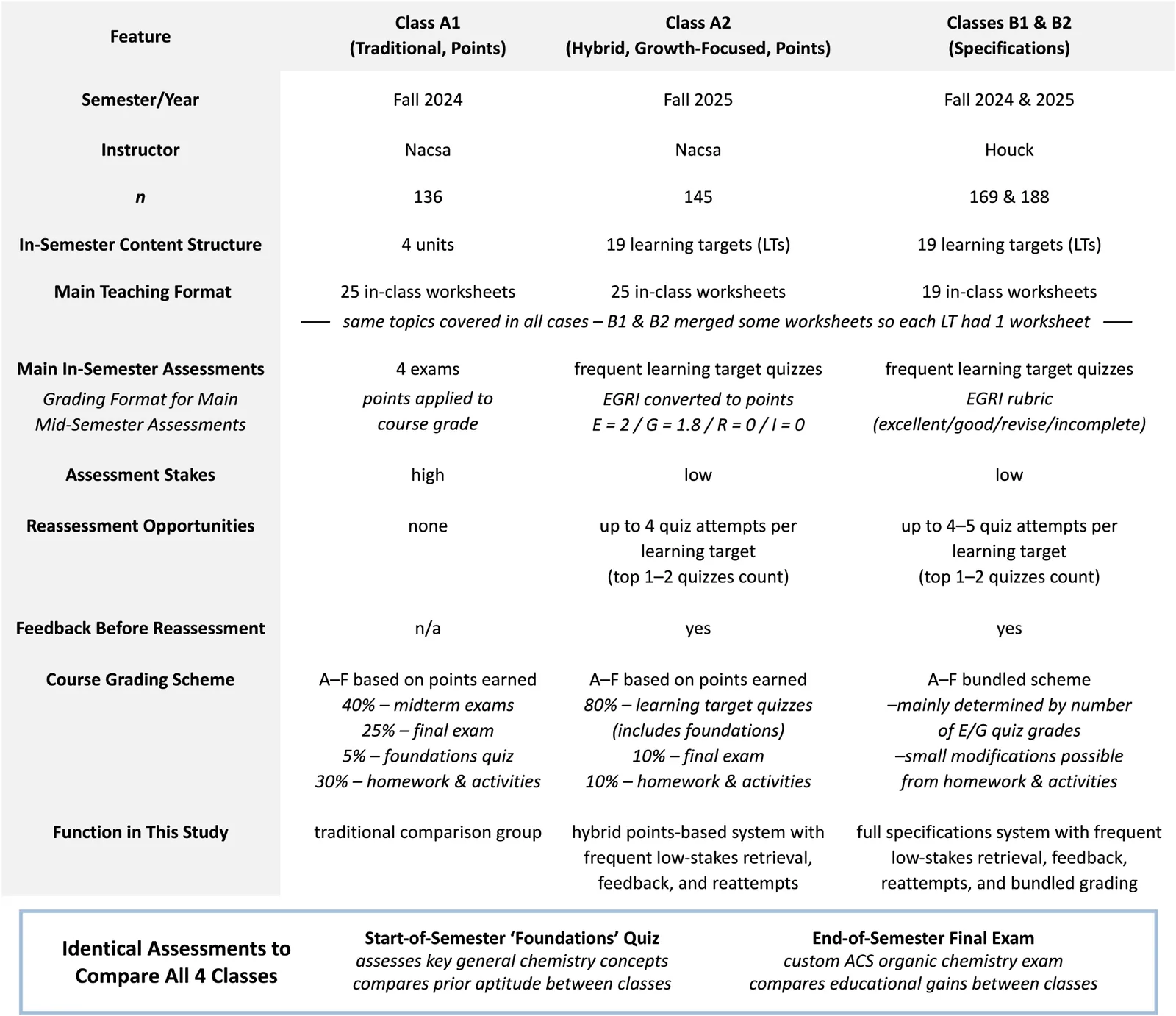
Study design over two years between two instructors and key features of course assessments and grading schemes.

### Ethical Considerations

This study was performed following The Pennsylvania State University Protocol for Human Subject Research (HRP-591). The protocol was reviewed and determined to be “research exempt” under STUDY00021800 by the university’s Human Research Protection Program, which serves as the Institutional Review Board for the institution. All study participants provided written informed consent.

### Institutional, Course, and Study Participant Context

All work was performed at Penn State’s flagship campus at University Park, in central Pennsylvania. Penn State is a large, state-related land-grant university with approximately 40,000 undergraduate students enrolled at this location. The student population is 53% male and 47% female; approximately 50% are Pennsylvania residents, 40% are from out-of-state, and 10% are international. The university is predominantly white (60%), with Hispanic (9%), Asian (7%), and Black (5%) students accounting for the next-largest racial/ethnic groups.

The classes involved in this study were four sections of Organic Chemistry I (CHEM 210) taught by two of the authors (J.D.H. and E.D.N.) during the Fall 2024 and Fall 2025 semesters. This course is the first of a two-semester sequence required for many science, engineering, and prehealth majors. During these years, our campus offered three sections of CHEM 210 each Fall, the semester when most students take this course (one “off-cycle” section was offered each Spring). Each section typically enrolled 200 to 250 students.

Among the 912 students who enrolled in the four sections, 638 provided written consent to participate in the study and completed both assessments used herein (70% participation rate, see Figure 2a for per-section details). The gender composition of each section ranged from 26–33% men and 66–73% women, with up to 5% expressing other gender identities or not contributing to the binary gender comparison (see Table S1 and the Demographic Survey in the Supporting Information, respectively, for gender data and full details of identity options provided to students). The overrepresentation of women in this course, relative to the overall university population, is consistent with longstanding enrollment patterns in this course and likely reflects the large number of prehealth students enrolled in Organic Chemistry I. Between 58% and 73% of students in each section identified exclusively as white, with Asian (11–21%), Hispanic (7–17%), and Black (4–9%) students forming the next-largest racial/ethnic groups, including students who selected multiple identities (see Table S2 in the Supporting Information).

To retain statistical power for subgroup analyses, students were grouped by gender identity (men and women) and race/ethnicity (“white only” students, who identified only with this racial or ethnic background, and “not white only” students, who selected at least one other identity, whether or not white was among their choices).

### Study Data

Gender identity and racial/ethnic background were determined from a survey completed as a course assignment. The relevant questions were “My gender or gender identity is best described as (select all that apply)” and “My racial or ethnic background is best described as (select all that apply).” The complete response options are provided in the Demographic Survey in the Supporting Information. These questions were part of a larger survey that included multiple affective measures.^20^ Analysis of the full survey was beyond the scope of this work.

Many students selected multiple racial/ethnic identities, but no students selected more than one gender option. Some students did not respond to all questions or did not identify as a man or a woman (the most common gender identities). Therefore, after listwise deletion of analyzed variables, the number of students included in subgroup analyses was slightly lower than the full analytic sample: 577 contributed to the man/woman gender comparison and to the race/ethnicity comparison. The number of students identifying outside the man/woman gender categories was too small for separate statistical analysis (*n* = 10). Although institutional gender data were also available for all students, survey responses were used instead to allow students to represent their gender identity more accurately.

Study participants consented to provide artifacts from this course, including quiz and exam scores, survey responses, and institutional student profile data. Students who withdrew from the course are underrepresented in the data set. As a result, DFW rates could not be meaningfully analyzed. Additionally, we did not have access to students’ GPAs, prior course grades, grades in other courses, or other institutional records beyond this course.

### Study Design

The four sections involved in this study were classified by instructor and year as shown in Figure 2. Sections taught by Instructor A (E.D.N.) were labeled A1 and A2, corresponding to Fall 2024 and Fall 2025, respectively. Sections taught by Instructor B (J.D.H.) were labeled B1 and B2, corresponding to Fall 2024 and Fall 2025, respectively. Instructor A was previously mentored in teaching by Instructor B, and both instructors sought to employ similar instructional styles that merged presenting information with active-learning strategies.

Class A1 used traditional points-based grading and served as the comparison section. In the following year, Instructor A taught a section similar in content and structure but replaced the traditional midterm exam structure with a hybrid, growth-focused assessment scheme that retained the familiar 100-point A–F scale; this section was labeled A2. Concurrently, Instructor B taught two sections, B1 and B2, that used full specifications grading.

This design allowed three comparisons addressing the study’s research questions. First, A1 and A2 provided a same-instructor comparison between traditional grading and a hybrid learning-target assessment structure. Second, A1 versus B1/B2 provided cross-instructor comparisons between traditional and full specs grading. Third, A2 versus B1/B2 allowed comparison of two implementations that shared frequent pass/fail learning-target quizzes, feedback, and reattempt opportunities but differed in how final course grades were determined. Specifically, A2 retained a points-based final-grade system, whereas B1/B2 used bundle-based specifications grading.

The hybrid system (A2) was designed to preserve the familiar 100-point course-grade structure while adopting several assessment features common to specs grading. This design allowed us to examine whether a more limited implementation focused on frequent, feedback-rich, pass/fail learning-target assessment with opportunities for reattempts produced outcomes similar to full specs grading without requiring the full transition to bundle-based final grades.

### Common Assessments across Sections

Two identical assessments were administered in all four sections. First, students completed a 25-question “Foundations Quiz” in the second week of the semester, before any substantial exposure to course-specific differences in grading structure. The Foundations Quiz was created by the instructors to assess prerequisite General Chemistry concepts determined to be most relevant to Organic Chemistry I, including Lewis structures, resonance, hybridization, geometry, polarity, and electronegativity. Items were multiple-choice or multiple-selection. This Foundations Quiz was used to compare incoming preparation across sections and, in subsequent analyses, to adjust or stratify ACS Organic Chemistry Exam scores.

Second, all students took the same 50-question final exam, which was a custom ACS Organic Chemistry First Term Exam.^29^ Questions were selected to align with the course learning objectives in structure, reactivity, and spectroscopy. The full syllabus for each section is provided in the Supporting Information. ACS exam scores served as the primary, externally validated outcome for comparing student performance across sections. The ACS Division of Chemical Education predicted a mean score of 28.0 (median score also 28.0) with a standard deviation of 6.5 for this 50-question custom exam based on per-question scores modeled from 4,278 students across 48 institutions. The recommended exam time was 80 min, although students in this study were given 60 min owing to scheduling constraints.

Distributions of final course grades are available in the Supporting Information (see Table S3 and Figure S1). They should be treated as descriptive data since they were not determined on a common scale among sections. It is also possible that A–F thresholds could be changed without affecting ACS exam results.

### Assessment and Grading Systems by Section

#### Traditional Points-Based Grading: A1

Class A1 used a traditional 100-point scale in which a student’s percentage course grade directly translated to an A–F letter grade. Most course points (70%) were earned from the Foundations Quiz (5%), four midterm exams (10% each; 40% total), and the final exam (25%). Students had one attempt per exam, and the points earned therefrom were added directly to their course grade. The remaining 30% of the grade came from in-class submissions, OWLv2 homework assignments, peer-led learning community sessions/recitations, and occasional participation-based activities (see Class A1 Syllabus in the Supporting Information for details). The course was divided into four units, each assessed by a midterm exam. Twenty-five in-class worksheets were used to deliver course content during 50-minute class sessions three times per week. This section served as the traditional points-based comparison group.

#### Hybrid Growth-Focused Learning-Target Assessments: A2

Class A2 used the same 25 in-class worksheets as Class A1. These worksheets remained as the primary course notes and occupied the majority of lecture time. The 100-point A–F scale was also retained, but the way students earned points was significantly modified.

The final exam, in-class activities, recitation attendance, and weekly homework were the same as in Class A1 and continued to directly contribute to the final course grade. However, the weight of these items was lowered from 55% in Class A1 to 20% in Class A2: 10% for the final exam and 10% for other assignments (in-class submissions, OWLv2 homework, recitations, and participation). The remaining 80% of the course grade came from Learning Target Quizzes (LTQs) designed to emphasize frequent low-stakes retrieval, feedback, and the opportunity to learn from mistakes. The four midterm exams in Class A1 were replaced with 19 Learning Targets (LTs). The 25 in-class worksheets were reorganized around these LTs, with each typically covering one or two worksheets. The Foundations Quiz served as the first LT.

Up to four attempts were offered for each LTQ. In most cases, two satisfactory LTQ scores for a given LT each contributed to 2% of the final course grade. The point value increased to 3% for quizzes associated with “essential” LTs and only one satisfactory score was counted for shorter LTs. Across the semester, 76 total LTQ attempts were available. To earn the full 80% of the quiz-based portion of the grade, students needed 33 satisfactory LTQ scores across the 19 LTs. See the Class A2 syllabus in the Supporting Information for additional details.

LTQs were graded using an EGRI rubric: Excellent, Good, Revision Needed, or Incomplete.^38^ A threshold for each EGRI category was specified on each quiz. In general, an Excellent score could be earned with a few minor errors per quiz, whereas a Good score required ∼75% correctness. Excellent and Good scores were considered satisfactory. In the hybrid points-based system, an Excellent score contributed full credit for that LTQ, a Good score contributed 90% credit, and Revision Needed or Incomplete scores contributed no credit.

Students in Class A2 began taking quizzes in the third week of the semester. The first attempt for each LTQ was offered shortly following completion of the material in class. The second attempt for all LTQs was offered approximately 1 week later, after the first attempt was scored and returned with feedback. These initial attempts were taken by the entire section at a common time each week in a single classroom listed in the course catalog as a required class meeting time, with each session proctored by two teaching assistants. Subsequent attempts for each LTQ were also provided at least 1 week after the previous attempt and were taken only by students who requested them to improve their earlier scores. Students satisfied with their initial scores could skip later attempts since no more than the top two LTQ scores counted toward the final grade, depending on the LT. Students signed up to take later attempts each week on an ad hoc basis that fit their schedules from a selection of common “quiz hours” using an online form. These sessions were offered in smaller classrooms (20–75-student capacities per section) and were proctored by 1–2 teaching assistants or undergraduate learning assistants.

This structure meant that students could earn full credit for the quiz-based portion of the course by earning satisfactory scores on early attempts and skipping later attempts. Conversely, students who initially earned Revision Needed or Incomplete scores could still earn full credit by demonstrating satisfactory performance on later attempts.

#### Full Specifications Grading: B1 and B2

Classes B1 and B2 used full specifications grading. Course content was organized around 19 LTs plus a cumulative final exam. Each LT had a clearly defined set of learning objectives such as structure and bonding, resonance, stereochemistry, acid–base chemistry, mechanisms, substitution and elimination reactions, spectroscopy, and chemistry case studies. Several targets were designated as “essential” because they reflected core concepts considered necessary for success in subsequent coursework.

Class time in B1 and B2 centered primarily on an analogous series of worksheets to those used in A1 and A2, although some worksheets were combined so that each LT was only associated with one worksheet. As in A2, student learning was assessed using short LTQs that were scored using the EGRI rubric. Excellent and Good scores were considered satisfactory.

Unlike A2, final course grades in B1 and B2 were not determined by accumulating points. Instead, a bundling structure was used wherein students demonstrated proficiency. An LT was considered proficient when a student earned a second satisfactory score on the same LT. Some shorter or integrative LTs were designated as one-time proficiency LTs and were considered both complete and proficient after a single satisfactory score. Additional requirements applied for grades of C or higher, including proficiency on essential LTs. Full bundling requirements are provided under the Class B1 and B2 syllabi in the Supporting Information.

Most LTQs could be attempted up to four or five times over the semester. Analogously to A2, 1–2 required initial attempts were administered at a common time, and subsequent optional attempts were offered at a selection of “quiz hours.”

In addition to LTQs, students completed a variety of lower-stakes assignments that supported practice and engagement. The same broad categories of lower-stakes practice activities were used across all four sections; these activities included in-class problems, OWLv2 homework and daily questions, peer-led learning community sessions, and occasional participation-based activities. These components did not independently determine final letter grades but instead contributed modestly through “experience point” (XP) modifications that could improve a student’s final completed or proficient LT totals. This design preserved the central role of demonstrated mastery of LTs while still rewarding consistent engagement and practice.

Although B1 and B2 differed from A2 in final course grade determination, all three modified sections shared several assessment features: clearly defined learning targets, frequent LTQs with satisfactory thresholds, feedback between attempts, repeated opportunities to demonstrate learning, and grading structures that reward eventual satisfactory performance rather than one-time performance.

### Data Analysis

Demographic survey results, Foundations Quiz scores, and ACS Organic Chemistry Exam scores were tabulated and plotted using Microsoft Excel. Statistical analyses were performed in R version 4.5.3.^39^

Primary analyses compared mean Foundations Quiz scores and ACS Organic Chemistry Exam scores across the four sections. One-way ANOVA was used for unadjusted comparisons across sections. When ACS exam scores were analyzed while accounting for incoming preparation, ANCOVA models were used with the Foundations Quiz as the covariate. The homogeneity-of-slopes assumption was evaluated for ANCOVA models. Because model diagnostics suggested some deviation from strict linearity between Foundations Quiz scores and ACS exam scores, additional sensitivity analyses grouped students into within-section Foundations Quiz tertiles representing lower, middle, and higher incoming preparation.

Effect sizes for omnibus ANOVA models were reported using ω^2^, with η^2^ values also calculated and reported in the Supporting Information. Effect sizes were interpreted using conventional thresholds: small (s) for ω^2^ > 0.01, medium (m) ω^2^ > 0.06, and large (l) for ω^2^ > 0.14.^40^ For ANCOVA models, partial ω^2^ values were reported where appropriate.

When omnibus tests indicated statistically significant differences among sections, post hoc pairwise comparisons were conducted using Tukey–Kramer tests to account for multiple comparisons among unequal group sizes. Pairwise effect sizes were reported using Cohen’s *d*, with Hedges’ *g* values also calculated and reported in the Supporting Information. Pairwise effects were interpreted using conventional thresholds: small (s) |*d*| > 0.2, medium (m) for |*d*| > 0.5, and large (l) for |*d*| > 0.8.^40^

Subgroup analyses examined whether section-level patterns were similar across students grouped by incoming preparation, gender identity, and racial/ethnic background. Students missing subgroup information were excluded only from the relevant subgroup analysis. Because some subgroup sample sizes were small, these analyses were interpreted as exploratory and were used to identify patterns warranting further study rather than to establish definitive subgroup-specific effects.

Unless otherwise stated, statistical significance was evaluated at *p* < 0.05. Tukey-adjusted *p*-values are reported for pairwise comparisons. In figures, asterisks correspond to *p* < 0.05 (*), *p* < 0.01 (**), and *p* < 0.001 (***). Error bars represent standard errors.

## Results

### Comparing Prior Preparation: Foundations Quiz

The focus of this study was to compare student learning across course sections that differed in assessment and grading structure. We first examined whether students entered the course with similar prerequisite preparation. This quiz assessed general chemistry concepts most relevant to the Organic Chemistry I course studied herein.

Mean Foundations Quiz scores were similar across the four sections. A one-way ANOVA indicated no statistically significant difference among sections: *F*(3, 634) = 0.78, *p* = 0.505, ω^2^ < 0.01, indicating a negligible effect (Figure 3a). Tukey–Kramer post hoc comparisons suggest that the sections did not differ in incoming preparation as measured by this course-specific instrument. However, Foundations Quiz scores were strongly associated with ACS Organic Chemistry Exam scores after accounting for course section, *F*(1, 633) = 291.16, *p* < 0.001, partial ω^2^ = 0.314, partial *r* = 0.56. Therefore, subsequent analyses accounted for incoming preparation in two ways: (1) by including Foundations Quiz scores as a covariate in ANCOVA models and (2) by grouping students within each course section into lower-, middle-, and higher-preparation tertiles based on Foundations Quiz scores.

**Figure 3:**
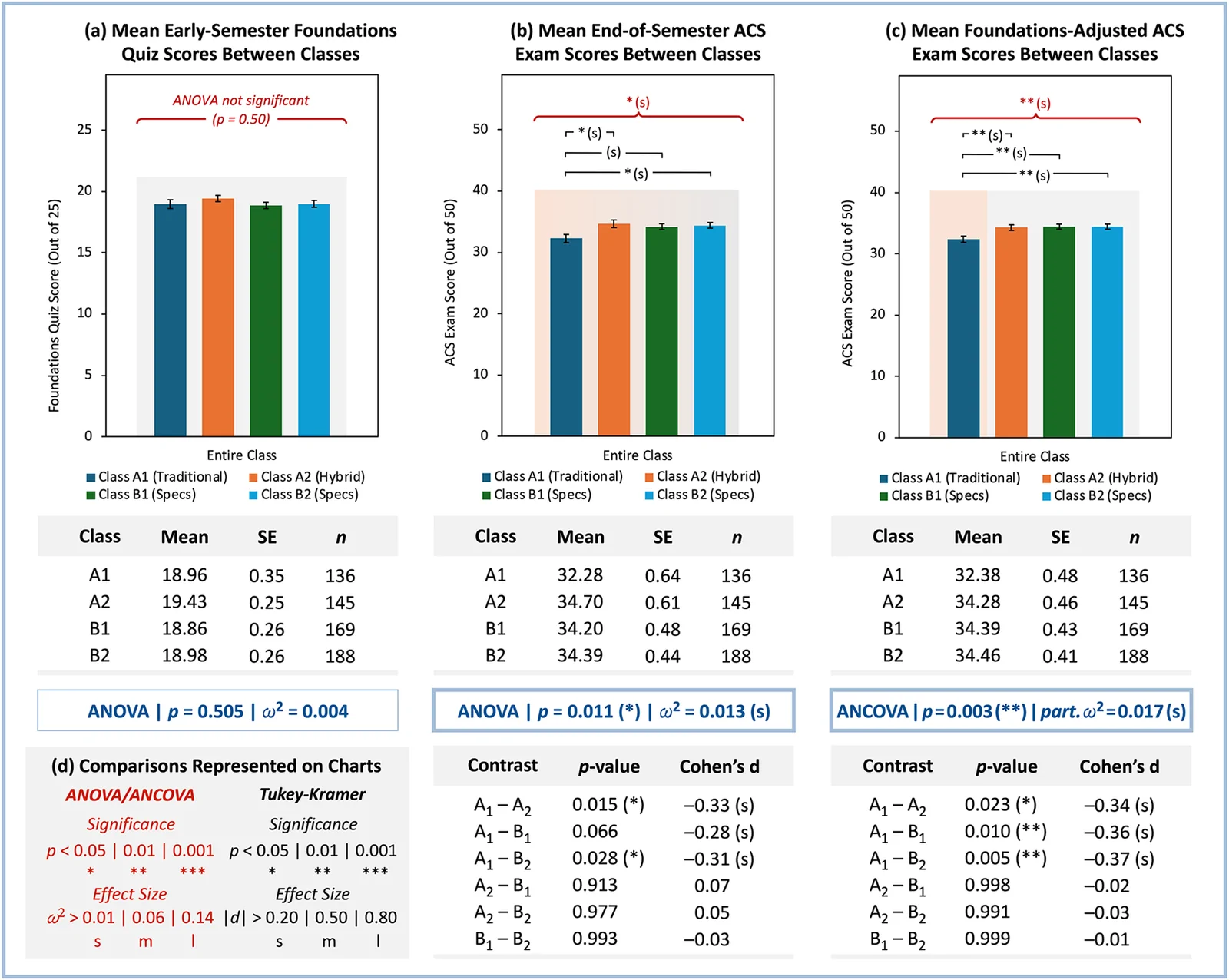
(a–c) Comparisons in “Foundations Quiz” scores, ACS Organic Chemistry Exam scores, and Foundations-Adjusted ACS Organic Chemistry Exam scores among classes. (d) Key for representations of statistical significance and effect sizes used herein. Error bars represent standard errors of the means (SE).

### Comparing Learning Gains: ACS Exam Performance

Student performance on the end-of-semester ACS Organic Chemistry Exam differed across sections (out of 50 points, Figure 3b). Mean ACS exam scores were lower for the traditionally graded section (A1, M = 32.28 ± 0.64, *n* = 136) than in the hybrid section (A2, M = 34.70 ± 0.61, *n* = 145) taught by the same instructor, and than in the full specs-graded sections (B1, M = 34.20 ± 0.48, *n* = 169 and B2, M = 34.39 ± 0.44, *n* = 188) taught by a different instructor.

ANOVA indicated a statistically significant difference among course sections, *F*(3, 634) = 3.72, *p* = 0.011, ω^2^ = 0.01, with a small effect size. Tukey–Kramer post hoc comparisons indicated that A1 scored significantly lower than A2 (*p* = 0.015, |*d*| = 0.33) and B2 (*p* = 0.028, |*d*| = 0.31). The difference between A1 and B1 was not statistically significant (*p* = 0.066), although the effect size was similar in magnitude (|*d*| = 0.28). No statistically significant pairwise differences were detected among the three modified-assessment sections, A2, B1, and B2 (*p* > 0.05), and all corresponding effect sizes were negligible (|*d*| < 0.07).

Because Foundations Quiz scores were strongly associated with ACS exam scores, we used ANCOVA to compare ACS exam performance while accounting for incoming preparation. In the additive ANCOVA model, Foundations Quiz score was a significant covariate, *F*(1, 633) = 291.16, *p* < 0.001, partial ω^2^ = 0.31. The homogeneity-of-slopes assumption was supported: the section by Foundations Quiz interaction was not statistically significiant, *F*(3, 630) = 1.26, *p* = 0.288, partial ω^2^ < 0.01. However, a sensitivity check indicated modest deviation from strict linearity, and adding a quadratic Foundations Quiz term improved the model fit, *F*(1, 632) = 4.92, *p* = 0.027. Therefore, we interpreted the ANCOVA results alongside the Foundations Quiz tertile analysis.

Adjusted mean ACS exam scores were lowest in A1. The ANCOVA indicated a statistically significant effect of section on ACS exam scores after adjusting for Foundations Quiz scores, *F*(3, 633) = 4.66, *p* = 0.003, partial ω^2^ = 0.02. Tukey-adjusted pairwise comparisons showed that A1 scored significantly lower than A2 (*p* = 0.023), B1 (*p* = 0.010), and B2 (*p* = 0.005). The corresponding standardized differences were small in magnitude: |*d*| = 0.34, |*d*| = 0.36, and |*d*| = 0.37, respectively. No significant differences were observed among A2, B1, and B2 (*p* > 0.05) and the corresponding standardized differences were negligible (|*d*| < 0.05).

Together, these analyses indicate that students using frequent, feedback-rich, pass/fail learning-target assessments with reattempts scored higher on the custom ACS Organic Chemistry Exam than students in the traditionally graded section. The similar outcomes observed in the hybrid points-based section and the full specs-graded sections suggest that the shared assessment structure, rather than the full bundle-based specs grading determination alone, may be a plausible contributor to the observed differences. These results, however, should be interpreted in light of the small overall effect sizes.

### Performance by Incoming Preparation

To examine whether the observed ACS exam score differences varied by incoming preparation, and to provide a sensitivity analysis for the ANCOVA model, students within each section were grouped into lower-, middle-, and higher-preparation tertiles based on Foundations Quiz scores. This treatment allowed us to test whether differences between the traditionally graded section (A1) and the modified-assessment sections (A2, B1, B2) were concentrated among the students entering the course with different levels of prerequisite preparation.

The differences between A1 and the modified sections were most pronounced among students in the lowest Foundations Quiz tertile (Figure 4a). Among these students, with the lowest incoming preparation, mean ACS exam scores were lower in A1 than in A2, B1, or B2. These differences were statistically significant, with medium pairwise effect sizes for all three comparisons of A1 vs modified sections. Similar but smaller patterns were observed among students in the middle Foundations Quiz tertile: A1 again had the lowest mean ACS exam score, and significant pairwise differences were observed between A1 and each of the modified sections with small or medium effects.

**Figure 4:**
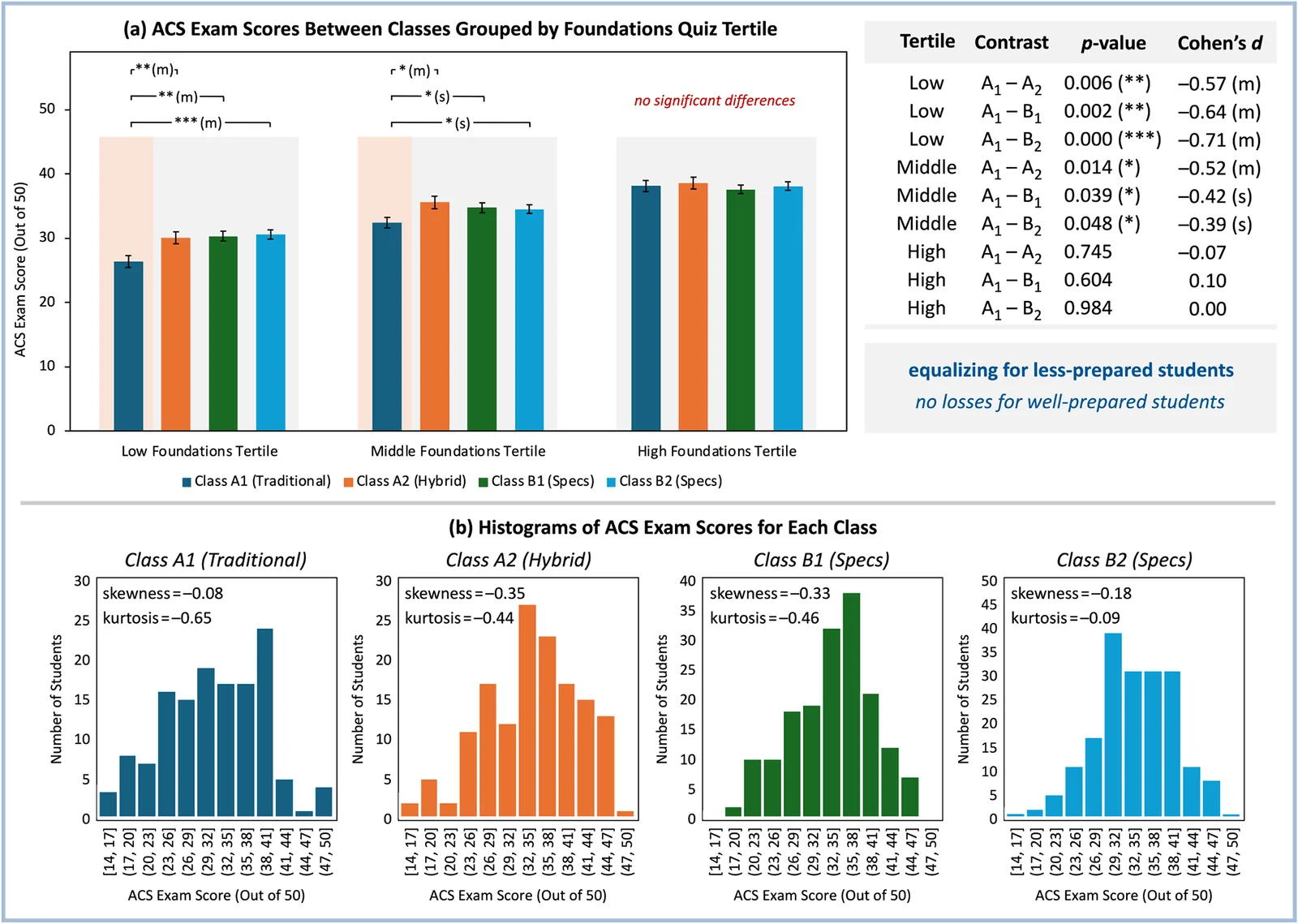
(a) Comparisons of mean ACS Organic Chemistry Exam scores among classes, sorted by within-class Foundations Quiz score tertile. Error bars represent standard errors of the means (SE). (b) Histograms of ACS Organic Chemistry Exam scores for each class.

In contrast, no statistically significant differences were observed between A1 and any of the modified sections among students in the highest Foundations Quiz tertile, and the corresponding effect sizes were negligible. Thus, the overall section-level differences in ACS exam performance appear to be concentrated primarily among students who entered the course with lower or moderate incoming preparation, with no evidence of corresponding losses among students with the highest incoming preparation.

The distributions of ACS exam scores were consistent with this interpretation (Figure 4b). The traditionally graded section (A1) had less negative skewness than the modified sections, indicating a less top-heavy distribution. Also, A1 had more negative kurtosis than A2, B1, or B2, reflecting a larger low-score tail. These distributional patterns are consistent with the tertile analysis; the modified sections appear to differ from the traditionally graded section most clearly among students in the lower portion of the incoming performance distribution.

### Performance by Gender Identity and Racial/Ethnic Background

Specs grading has been proposed as one way to reduce inequities associated with traditional high-stakes assessment.^13,33–37^ Recent chemistry education research has begun to examine this possibility, with mixed results.^10^ Therefore, we examined whether ACS exam performance patterns differed across student subgroups defined by gender identity and racial/ethnic background. Because incoming preparation was associated with ACS exam scores, these subgroup analyses used Foundations-adjusted ACS exam scores. For students identifying as men, Foundations-adjusted ACS exam scores did not differ significantly across course sections, *F*(3, 163) = 1.86, *p* = 0.139, partial ω^2^ = 0.02 (Figure 5a). Tukey-adjusted pairwise comparisons also did not identify significant differences among sections for this subgroup (*p* > 0.05). For students identifying as women, Foundations-adjusted ACS exam scores did differ significantly across course sections, *F*(3, 404) = 4.57, *p* = 0.004, partial ω^2^ = 0.03. Tukey-adjusted pairwise comparisons indicate that A1 scored significantly lower than B1 (*p* = 0.030, |*d*| = 0.40) and B2 (*p* = 0.002, |*d*| = 0.51). The A1 versus A2 comparison was not significant (*p* = 0.218), but the direction of the difference was consistent with the overall pattern. No statistically significant differences were detected among A2, B1, and B2 (*p* > 0.05).

**Figure 5:**
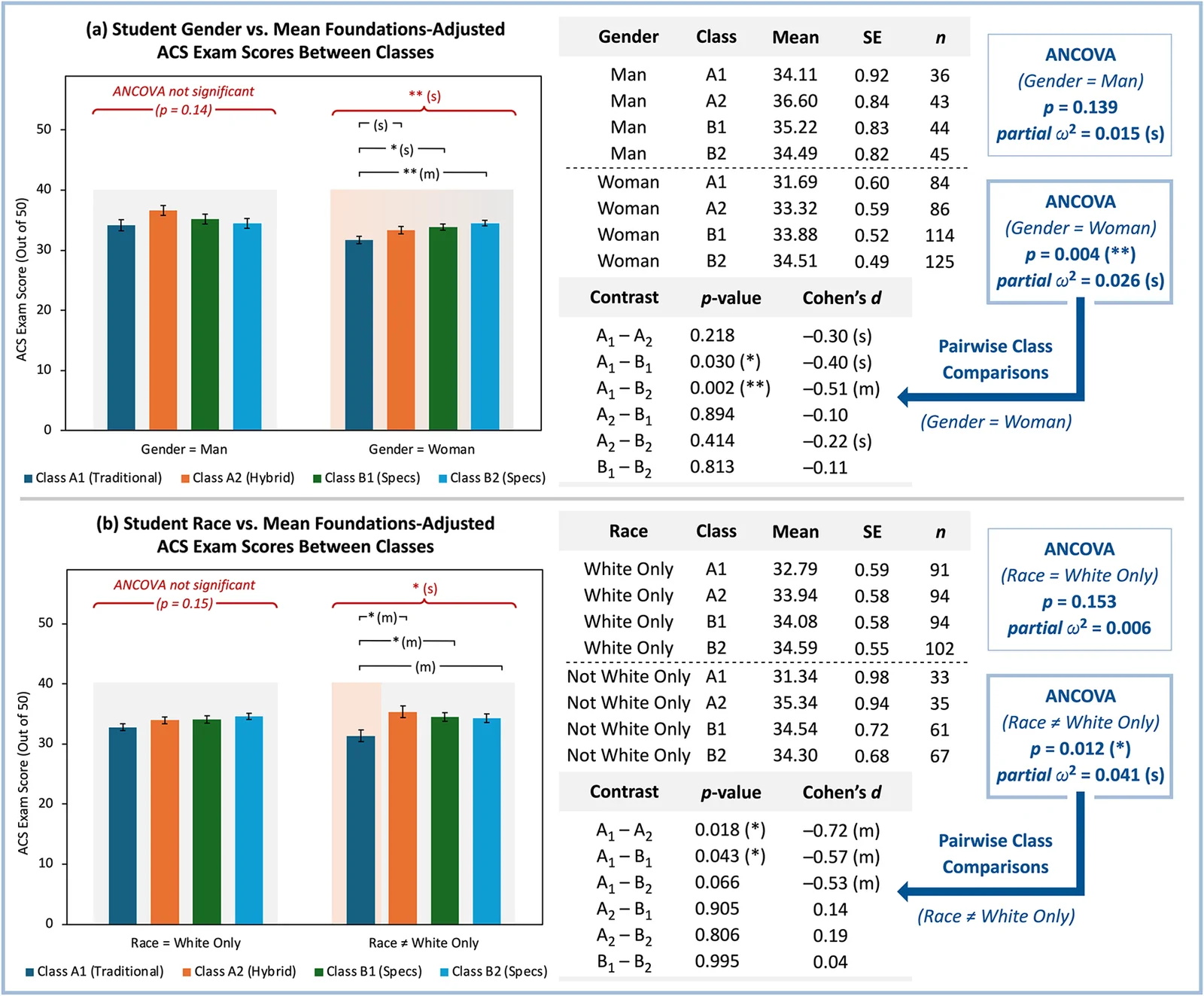
Comparisons of mean Foundations-adjusted ACS Organic Chemistry Exam scores between classes subdivided by (a) gender identity and (b) race/ethnicity. Error bars represent standard errors of the means (SE).

Patterns by racial/ethnic background were similar (Figure 5b). Among students who identified exclusively as white, Foundations-adjusted ACS exam scores did not differ significantly across course sections, *F*(3, 376) = 1.77, *p* = 0.153, partial ω^2^ < 0.01. Among students who did not identify exclusively as white, Foundations-adjusted ACS exam scores did differ significantly across course sections, *F*(3, 191) = 3.77, *p* = 0.012, partial ω^2^ = 0.04. Tukey-adjusted pairwise comparisons indicate that A1 also scored significantly lower than A2 (*p* = 0.018, |*d*| = 0.72) and B1 (*p* = 0.043, |*d*| = 0.57), but not significantly lower than B2 (*p* = 0.066, |*d*| = 0.54); however, all comparisons had medium effect sizes. No statistically significant differences were detected among A2, B1, and B2 (*p* > 0.05).

To test whether these subgroup patterns reflected different section effects across social identity groups, we fit interaction models that included section, subgroup, Foundations Quiz score, and section-by-subgroup interaction. The section-by-gender identity interaction was not statistically significant, *F*(3, 568) = 2.05, *p* = 0.106, partial ω^2^ < 0.01, indicating that the evidence did not support a statistically different section effect for students identifying as men versus women. Likewise, the section-by-race/ethnicity interaction was not statistically significant, *F*(3, 568) = 1.19, *p* = 0.311, partial ω^2^ < 0.01, also indicating that the evidence did not support a statistically different section effect for students who identified exclusively as white versus those who do not. Thus, these subgroup analyses suggest that the section-level differences were more apparent among students identifying as women and among students who did not identify exclusively as white, but they do not establish that the modified assessment structures had statistically different effects across gender or racial/ethnic groups. These findings should be interpreted as exploratory and hypothesis-generating rather than as definitive evidence of reduced social identity performance gaps.

## Discussion

Beyond the main benefits of specifications grading originally hypothesized by Nilson, these results provide new evidence that assessment structures can promote tangible educational learning gains. The central finding of this study is that students in courses using frequent, low-stakes, pass/fail learning target-focused assessment with feedback and reattempt opportunities scored higher on a custom ACS Organic Chemistry Exam than students in a traditionally graded section organized around high-stakes midterm exams. This pattern was observed in both unadjusted analyses and those that accounted for incoming preparation using Foundations Quiz scores. Although the overall effect sizes were small, the consistency of the A1 versus modified section contrasts across unadjusted and adjusted analyses supports the finding that modified assessment structures were associated with higher performance on the same exam.

Importantly, similar ACS exam outcomes were observed in the hybrid points-based section and the two full specs-grading sections. The hybrid section retained a traditional 100-point course structure, whereas the full specs grading sections used a bundling system to determine final course grades. However, all three modified sections shared several assessment features: clearly defined learning targets, frequent lower-stakes pass/fail quizzes, feedback between attempts, and opportunities to reattempt quizzes. The absence of meaningful differences among the hybrid and full specs grading sections suggests that this assessment structure may be a more plausible contributor to the observed ACS exam differences than the full bundling system used in specs grading.

This interpretation aligns with broader literature on retrieval practice, feedback, and low-stakes testing.^30–32^ This study contributes course-scale evidence from large-enrollment organic chemistry that an assessment system built around these features can be associated with improved performance on an externally validated assessment. Therefore, the results support a narrower and more mechanistic claim than “specifications grading improves learning”; rather, results suggest that frequent, feedback-rich, pass/fail LT quizzes with reattempts may be one important mechanism through which specifications-style and related alternative grading systems can support student learning.

The preparation-tertile analysis clarifies the nature of the observed differences. The largest differences between the traditionally graded section and the modified-assessment sections occurred among students in the lowest Foundations Quiz tertile, with smaller differences among students in the middle tertile and no detectable differences among students in the highest tertile. This pattern suggests that modified assessment structures were associated with improved ACS exam performance primarily among students entering the course with lower or moderate preparation. Additionally, there was no evidence that students entering with the highest preparation performed worse in those modified sections, which suggests that the observed gains were not accompanied by detectable losses among students who entered the course with stronger prerequisite preparation.

The subgroup analyses are consistent with the possibility that assessment structures may support students who are less well served by traditional high-stakes exams. The subgroup analyses by gender identity and racial/ethnic background were broadly consistent with the preparation-tertile analysis. Adjusted ACS exam differences were more apparent among students identifying as women and among students who did not identify exclusively as white. These results are consistent with the hypothesis that lower-stakes, feedback-rich, reattemptable assessment structures may support students who are less well served by traditional high-stakes exams. However, these findings should be interpreted cautiously as the subgroup analyses were exploratory and some subgroup sample sizes were limited. Formal interaction tests did not detect significant differences in section effects across gender identity or racial/ethnic subgroups after adjusting for Foundations Quiz scores. Therefore, these findings suggest that modified assessment structures may lead to reduced social identity performance gaps and warrant further investigation.^10^

Several features may explain why frequent learning target assessments with feedback and reattempts were associated with higher ACS exam performance. First, distributing assessments across many LTQs with reattempt opportunities meant that an initially poor performance did not permanently determine credit; instead, students could continue engaging with the same learning target and demonstrate improvement on a later attempt. This repeated engagement may have provided additional opportunities to strengthen initially weak topics before the cumulative ACS exam. Second, the targeted scope of each LTQ may have helped students focus their studying on specific concepts and skills. Third, feedback between attempts gave students opportunities to identify and correct alternative conceptions before later assessments. Fourth, requiring students to reach a satisfactory threshold may have encouraged attention to core learning objectives rather than partial-credit accumulation. While this study design cannot disentangle which features were most important, future studies should manipulate these elements more directly to isolate the relative contributions of assessment frequency, feedback, reattempt opportunities, assessment stakes, satisfactory thresholds, and final grade determination.^41^

## Limitations

Several considerations limit these conclusions. First, the study was quasi-experimental, as students were not randomly assigned to a grading condition. Although same-instructor comparisons and analyses that accounted for incoming preparation were performed, unmeasured differences across sections may have contributed to the observed outcomes.

Second, the study was conducted in one large-enrollment Organic Chemistry I course at a single institution. It is unclear how these findings generalize to smaller courses, other chemistry subdisciplines, other institutions, or student populations with different demographics and academic profiles.

Third, the Foundations Quiz was instructor-created and course-specific rather than an instrument with external validation in other contexts. It was designed to measure prerequisite general chemistry concepts important for this specific Organic Chemistry I course, and it was strongly associated with ACS exam performance in this data set. The use of the Foundations Quiz scores as a covariate and tertile-grouping variable helps address some concerns about baseline preparation, but it does not account for all possible preexisting differences among students or sections.

Fourth, DFW grades could not be meaningfully analyzed. Alternative grading systems, like specs grading, may not only affect exam performance among students who complete the course, but also persistence, withdrawal, and course completion.

Fifth, subgroup analyses were limited by available social identity data and sample size. Missing subgroup classification data reduced sample sizes, particularly for gender identity and race/ethnicity, and participants who did not provide usable responses may differ from those who did. Some groups, including students identifying outside of the man/woman binary, were too small for statistical analysis. Additionally, race/ethnicity was simplified into “identify exclusively as white” and “did not identify exclusively as white” to preserve statistical power, which collapses differences among students’ identities and experiences. Accordingly, subgroup analyses should be interpreted as exploratory patterns rather than definitive estimates of effects for specific subgroups.

Finally, the study cannot isolate the independent effects of retrieval practice, assessment frequency, feedback, reattempt opportunities, assessment stakes, satisfactory thresholds, and final grade determination. The similarity between the hybrid and full specs grading sections suggests that the shared assessment structure is likely important, but future studies should manipulate these features to determine their relative importance.

## Implications

For instructors, findings suggest that shifting assessment away from a small number of high-stakes exams and toward frequent, feedback-rich, reattemptable LT assessments may support student performance on externally validated exams. Importantly, the hybrid points-based section produced similar outcomes to the full specs grading sections, suggesting that instructors interested in alternative grading may not need to begin by replacing the entire course grading system.^12^ Instead, a more focused change to the assessment structure may offer a practical starting point.

For students, the preparation-tertile analysis suggests that the benefits of this assessment structure may be most relevant for students entering the course with lower or moderate prerequisite preparation. These students showed the clearest ACS exam differences between the traditionally graded section and the modified-assessment sections. Notably, students in the highest-preparation tertile showed no detectable losses. Student-facing explanations of the course structure should emphasize how repeated LT assessments, feedback, and reattempts are intended to support improvement over time rather than merely increasing the number of scored assessments.^17^

For departments and administrators, findings suggest that support for alternative assessment should not only focus on full specs grading implementations. Because the hybrid and full specs grading sections produced similar outcomes, departments may consider supporting intermediate models that retain familiar points-based course grades while adopting frequent LT assessment with feedback and reattempts. Such support should recognize instructional innovations and provide instructors with instructional support, such as teaching assistantships or course releases, to support assessment reform.

For researchers, findings reinforce the need to study alternative grading systems at the level of specific assessment features rather than only as whole-course grading systems. The similar performance of the hybrid and full specs grading sections suggests that the shared assessment structure may be a key contributor to the observed ACS exam score differences. However, this study design cannot isolate the independent effects of the assessment structure. Future research should compare targeted variants of these structures with precourse preparation measures, standardized learning outcomes, and adequate statistical power to examine whether effects differ across student groups.

## Conclusion

This quasi-experimental study provides evidence that replacing high-stakes exams with frequent, feedback-rich, pass/fail learning-target-focused assessments with reattempts was associated with higher scores on a custom ACS Organic Chemistry Exam in large-enrollment Organic Chemistry I lectures. Similar outcomes in the hybrid and full specifications grading sections suggest that the shared assessment structure may have contributed to the observed gains. Improvements were concentrated among students entering with lower or moderate preparation, with no detectable losses among higher-preparation students. Exploratory demographic subgroup patterns were consistent with this trend. Future work should isolate which features of specifications-style assessment most strongly support student learning.

## Supplementary Material











## Notes

Demographic survey questions, course demographics, and tabulated results of statistical tests (PDF)
Class A1 syllabus (PDF)
Class A2 syllabus (PDF)
Class B1 syllabus (PDF)
Class B2 syllabus (PDF)
