# Supplementary material for: Learning Gains in Organic Chemistry from Full and Hybrid Specifications Grading
Source: JACS Au. 2026 Sep 3;6(9):4956–67. doi: 10.1021/jacsau.6c00531 (PMC13625744; doi:10.1021/jacsau.6c00531)
Supplement: Supplementary file 1 [file au6c00531_si_001.pdf]

Supporting Information for

Learning Gains in Organic Chemistry From Full and Hybrid Specifications Grading

Joseph D. Houck,[1]* Brandon J. Yik,[2]* and Eric D. Nacsa[1]*

[1] Department of Chemistry, The Pennsylvania State University, University Park, PA 16802, United States

[2] Department of Chemistry, University of Georgia, Athens, GA 30602, United States

** corresponding author emails: jdh68@psu.edu, byik@uga.edu, nacsa@psu.edu*

TABLE OF CONTENTS

I. Demographic Survey Questions S3

II. Course Data and Results of Statistical Tests S4

I. Demographic Survey Questions

What is your major?

- o ____________________

My gender or gender identity is best described as (select all that apply):

- o I am agender
- o I am a man
- o I am nonbinary
- o I am gender questioning
- o I am gender nonconforming
- o I am genderqueer or genderfluid
- o I am questioning
- o I am transgender
- o I am a woman
- o My gender or gender identity is best described as: ____________________
- o I prefer not to disclose my gender or gender identity

My racial or ethnic background is best described as (select all that apply):

- o Black, Afro-Caribbean, or African American (e.g., Jamaican, Nigerian, Haitian, Ethiopian, etc.)
- o Asian or Asian American (e.g., Chinese, Japanese, Filipino, Korean, South Asian, Vietnamese, etc.)
- o Indigenous American or Alaska Native (e.g., Navajo Nation, Blackfeet Tribe, Inupiat Traditional Gov't., etc.)
- o Latino/a/e/x or Hispanic American (e.g., Puerto Rican, Mexican, Cuban,Salvadoran, Colombian, etc.)
- o Middle Eastern, North African, or Arab American (e.g., Lebanese, Iranian, Egyptian, Moroccan, Israeli, Palestinian, etc.)
- o Native Hawai'ian or Pacific Islander (e.g., Samoan, Guamanian, Chamorro,Tongan, etc.)
- o Non-Hispanic White or Euro-American (e.g., German, Irish, English, Italian, Polish, French, etc.)
- o My race or ethnicity is best described as: ____________________
- o I prefer not to disclose my racial or ethnic background.

II. Course Data and Results of Statistical Tests

Table S1. Gender Composition of Classes

Class	A1	A2	B1	B2
Usable Responses	126	130	159	172
Man	36	43	44	45
Woman	84	86	114	125
Other	6	1	1	2
% Man	28.6	33.1	27.7	26.2
% Woman	66.7	66.2	71.7	72.7
% Other	4.8	0.8	0.6	1.2

Table S2. Race Composition of Classes

Class	A1	A2	B1	B2	Specific Survey Selection
Usable Responses	124	132	163	171	
White Only	91	94	94	102	"Non-Hispanic White or Euro-American" as only selection
Not White Only	33	35	61	67	not only "Non-Hispanic White or Euro-American"
Black	10	5	14	11	"Black, Afro-Caribbean, or African American" & any other selection
Asian	13	25	34	35	"Asian or Asian American" & any other selection
Native American	1	0	3	1	"Indigenous American or Alaska Native" & any other selection
Hispanic	8	9	28	14	"Latino/a/e/x or Hispanic American" & any other selection
Middle Eastern, Arab, or North African	2	2	3	7	"Middle Eastern, North African, or Arab American" & any other selection
Native Hawai'ian or Pacific Islander	0	0	2	2	"Native Hawai'ian or Pacific Islander" & any other selection
% White Only	73.4	71.2	57.7	59.6	
% Non-(White Only)	26.6	26.5	37.4	39.2	
% Black	8.1	3.8	8.6	6.4	
% Asian	10.5	18.9	20.9	20.5	
% Native American	0.8	0.0	1.8	0.6	
% Hispanic	6.5	6.8	17.2	8.2	
% Middle Eastern, Arab, or North African	1.6	1.5	1.8	4.1	
% Native Hawai'ian or Pacific Islander	0.0	0.0	1.2	1.2	

Table S3. Final Grade Distributions of Classes

Class	A1	A2	B1	B2
n	136	145	169	188
A	73	90	121	133
B	28	34	25	30
C	31	16	10	11
D	3	2	10	10
F	1	3	3	4
% A	53.7	62.1	71.6	70.7
% B	20.6	23.4	14.8	16.0
% C	22.8	11.0	5.9	5.9
% D	2.2	1.4	5.9	5.3
% F	0.7	2.1	1.8	2.1

Figure S1. Final Grade Distributions of Classes

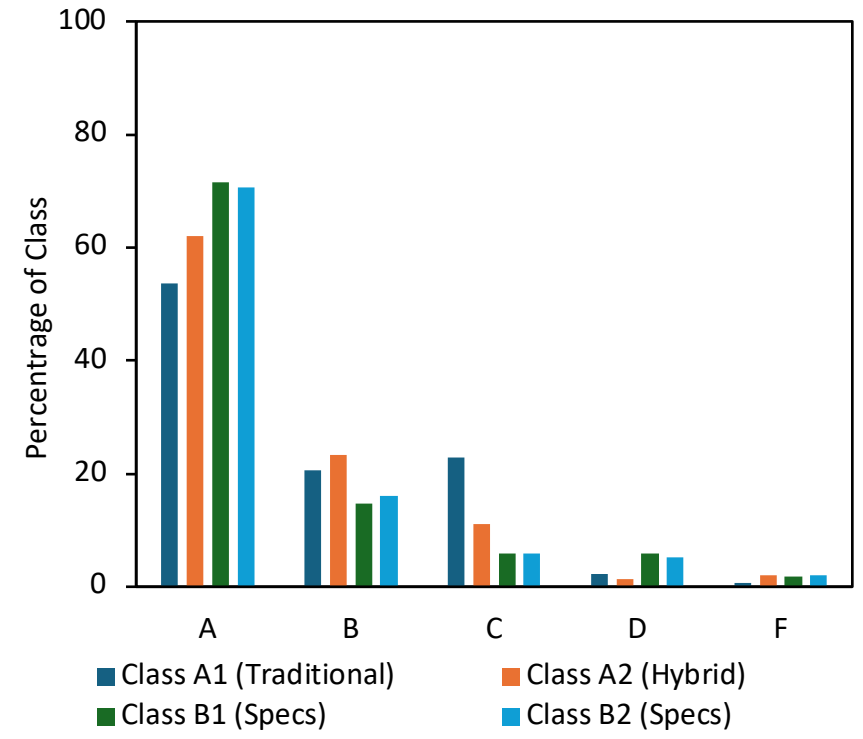

Note: Final grade distributions are provided descriptively only and were not used as the primary learning outcome because grading systems differed across sections.

Table S4. Summary Statistics for Common Assessments by Class

Assessment	Class	*n*	Mean	SE	Median	Min	Max	Skewness	Kurtosis
Foundations Quiz	A1	136	18.9574	0.3494	19.58	5.75	25	-0.9571	0.5230
Foundations Quiz	A2	145	19.4343	0.2534	19.75	9.08	24.25	-0.8477	0.8432
Foundations Quiz	B1	169	18.8625	0.2611	19.5	7.25	24.75	-0.8560	0.5631
Foundations Quiz	B2	188	18.9831	0.2627	19.7	5.7	24.1	-0.9510	0.6766
ACS Exam	A1	136	32.2794	0.6440	32.5	17	50	-0.0837	-0.6520
ACS Exam	A2	145	34.6966	0.6085	35	17	48	-0.3547	-0.4389
ACS Exam	B1	169	34.1953	0.4791	35	20	46	-0.3330	-0.4648
ACS Exam	B2	188	34.3936	0.4446	34	15	49	-0.1819	-0.0924

Table S5. ANOVA for Common Assessments Among Classes

Assessment	n_{total}	n_{groups}	df	df_{error}	ss_{effect}	ss_{error}	Statistic	*p*-value	η^2	ω^2	Effect Size	Significance
Foundations Quiz	638	4	3	634	29.3635	7943.6622	0.7812	0.5047	0.003683	0.000000	negligible	
ACS Exam	638	4	3	634	507.4813	28811.4592	3.7224	0.0113	0.017309	0.012639	small	*

Table S6. Pairwise Comparisons for Common Assessments Between Classes

Assessment	Comparison	n_1	n_2	M_1	M_2	$M_2 - M_1$	SD_{pooled}	*p*-value	Cohen's *d*	Hedges' *g*	Effect Size	Significance
Foundations Quiz	A2-A1	136	145	18.9574	19.4343	0.4769	3.5833	0.6719	0.1331	0.1327	negligible	
Foundations Quiz	B1-A1	136	169	18.9574	18.8625	-0.0949	3.7128	0.9956	-0.0256	-0.0255	negligible	
Foundations Quiz	B2-A1	136	188	18.9574	18.9831	0.0257	3.8074	0.9999	0.0067	0.0067	negligible	
Foundations Quiz	B1-A2	145	169	19.4343	18.8625	-0.5718	3.2403	0.4829	-0.1765	-0.1760	negligible	
Foundations Quiz	B2-A2	145	188	19.4343	18.9831	-0.4513	3.3734	0.6564	-0.1338	-0.1335	negligible	
Foundations Quiz	B2-B1	169	188	18.8625	18.9831	0.1205	3.5051	0.9885	0.0344	0.0343	negligible	
ACS Exam	A2-A1	136	145	32.2794	34.6966	2.4171	7.4164	0.0147	0.3259	0.3250	small	*
ACS Exam	B1-A1	136	169	32.2794	34.1953	1.9159	6.8294	0.0661	0.2805	0.2798	small	
ACS Exam	B2-A1	136	188	32.2794	34.3936	2.1142	6.7254	0.0281	0.3144	0.3136	small	*
ACS Exam	B1-A2	145	169	34.6966	34.1953	-0.5013	6.7575	0.9131	-0.0742	-0.0740	negligible	
ACS Exam	B2-A2	145	188	34.6966	34.3936	-0.3029	6.6595	0.9773	-0.0455	-0.0454	negligible	
ACS Exam	B2-B1	169	188	34.1953	34.3936	0.1984	6.1588	0.9925	0.0322	0.0321	negligible	

Table S7. Mean ACS Exam Scores Adjusted for Foundations Quiz Scores by Class

Class	Estimate	SE	*df*	Statistic	*p*-value
A1	32.3765	0.4788	633	67.6175	0
A2	34.2838	0.4643	633	73.8370	0
B1	34.3938	0.4297	633	80.0487	0
B2	34.4632	0.4072	633	84.6261	0

Table S8. ANCOVA Type 2 for Foundations Quiz-Adjusted ACS Exam Scores Among Classes

Model	Term	*df*	df_{error}	ss_{effect}	ss_{error}	Statistic	*p*-value	Partial η^2	Partial ω^2	Effect Size	Significance
ANCOVA: ACS ~ Class + Foundations	Class	3	633	435.5993	19734.3466	4.6574	0.003144	0.021596	0.016933	small	**
ANCOVA: ACS ~ Class + Foundations	Foundations	1	633	9077.1126	19734.3466	291.1580	0.000000	0.315052	0.313631	large	***
ANCOVA: ACS ~ Class + Foundations	Residuals	633	633	19734.3466	19734.3466	NA	NA	0.5	0	negligible	

Table S9. ANCOVA Type 3 Homogeneity Interaction

Model for Moderation Check	Term	*df*	df_{error}	ss_{effect}	ss_{error}	Statistic	*p*-value	Partial η^2	Partial ω^2	Effect Size	Significance
ACS ~ Class * Foundations	Class	3	630	183.5281	19616.8387	1.9647	0.118042	0.009269	0.004544	negligible	
ACS ~ Class * Foundations	Foundations	1	630	8852.6899	19616.8387	284.3065	0.000000	0.310953	0.309521	large	***
ACS ~ Class * Foundations	Class: Foundations	3	630	117.5078	19616.8387	1.2579	0.287922	0.005954	0.001219	negligible	
ACS ~ Class * Foundations	Residuals	630	630	19616.8387	19616.8387	NA	NA	0.500000	0.000000	negligible	

Table S10. ANCOVA Linearity Quadratic Check

Term	$df_{residuals}$	*rss*	*df*	*ss*	Statistic	*p*-value	Comparison	Significance
ACS ~ Class + Foundations	633	19734.3466	NA	NA	NA	NA	Add quadratic Foundations term	NA
ACS ~ Class + Foundations + I(Foundations²)	632	19581.9523	1	152.3942	4.9185	0.0269	Add quadratic Foundations term	*

Table S11. Pairwise Comparisons of Foundations Quiz-Adjusted ACS Exam Scores Between Classes (Tukey-Unadjusted *p*-Values)

Comparison	Difference	SE	Standardized Difference	Standardized SE	*df*	Statistic	*p*-value	Significance	Cohen's *d*	Effect Size
A1 - A2	-1.9073	0.6672	-0.3416	0.1199	633	-2.8496	0.004520	**	-0.34	small
A1 - B1	-2.0173	0.6432	-0.3613	0.1156	633	-3.1241	0.001865	**	-0.36	small
A1 - B2	-2.0868	0.6285	-0.3737	0.1131	633	-3.3057	0.001001	**	-0.37	small
A2 - B1	-0.1099	0.6331	-0.0197	0.1134	633	-0.1737	0.862174		-0.02	negligible
A2 - B2	-0.1794	0.6178	-0.0321	0.1106	633	-0.2905	0.771555		-0.03	negligible
B1 - B2	-0.0695	0.5919	-0.0124	0.1060	633	-0.1174	0.906572		-0.01	negligible

Table S12. Foundations Quiz Tertile Boundaries by Class

Class	Tertile	*n*	Min	Max	Mean	SD
A1	Low	46	5.75	18.5	14.3100	3.1867
A1	Middle	45	18.58	21.25	19.8262	0.8369
A1	High	45	21.25	25	22.8393	1.0470
A2	Low	49	9.08	18.5	16.0382	2.2620
A2	Middle	48	18.58	21	19.8285	0.8094
A2	High	48	21	24.25	22.5071	0.9044
B1	Low	57	7.25	18	14.9691	2.4445
B1	Middle	56	18.08	20.75	19.4839	0.8349
B1	High	56	20.75	24.75	22.2041	0.9656
B2	Low	63	5.7	18.05	14.8149	2.6877
B2	Middle	63	18.1	21.3	19.7592	0.9791
B2	High	62	21.33	24.1	22.4298	0.9030

Table S13. Summary Statistics for ACS Exam Scores by Class Subdivided by Foundations Quiz Tertiles

Class	Tertile	*n*	Mean	SD	SE	Median	Min	Max	Skewness	Kurtosis
A1	Low	46	26.4130	6.2310	0.9187	26	17	41	0.4091	-0.6306
A1	Low	49	30.0816	6.4770	0.9253	30	17	42	-0.1616	-0.7601
A1	Low	57	30.2982	5.8675	0.7772	31	20	42	-0.0046	-1.0595
A2	Low	63	30.6190	5.6893	0.7168	31	15	42	-0.2778	-0.2583
A2	Middle	45	32.4222	5.5370	0.8254	32	19	42	-0.2640	-0.5083
A2	Middle	48	35.5625	6.5230	0.9415	36	18	46	-0.2812	-0.3456
B1	Middle	56	34.7679	5.6440	0.7542	35	20	45	-0.4134	-0.1794
B1	Middle	63	34.5079	5.1833	0.6530	34	22	47	0.0991	-0.6162
B1	High	45	38.1333	5.6270	0.8388	39	26	50	-0.1743	-0.0644
B2	High	48	38.5417	6.4046	0.9244	39	17	48	-0.9383	1.1062
B2	High	56	37.5893	4.8758	0.6516	38	23	46	-0.3066	0.1248
B2	High	62	38.1129	4.9921	0.6340	38	26	49	-0.0005	-0.5608

Table S14. Pairwise Comparisons of ACS Exam Scores Between Class A1 and Other Classes Subdivided by Foundations Quiz Tertiles

Class	Tertile	n_{A1}	$n_{treatment}$	M_{A1}	$M_{treatment}$	$M_{treatment} - M_{A1}$	SE	Statistic	*p*-value	Cohen's *d*	Hedges' *g*	Effect Size	Significance
A2	Low	46	49	26.4130	30.0816	3.6686	1.3055	2.8101	0.006039	0.576898	0.572233	medium	**
A2	Middle	45	48	32.4222	35.5625	3.1403	1.2587	2.4948	0.014408	0.517660	0.513382	medium	*
A2	High	45	48	38.1333	38.5417	0.4083	1.2535	0.3257	0.745365	0.067592	0.067033	negligible	
B1	Low	46	57	26.4130	30.2982	3.8852	1.1956	3.2497	0.001570	0.644081	0.639287	medium	**
B1	Middle	45	56	32.4222	34.7679	2.3456	1.1204	2.0935	0.038862	0.419112	0.415929	small	*
B1	High	45	56	38.1333	37.5893	-0.5440	1.0456	-0.5203	0.604018	-0.104163	-0.103372	negligible	
B2	Low	46	63	26.4130	30.6190	4.2060	1.1487	3.6614	0.000392	0.710091	0.705102	medium	***
B2	Middle	45	63	32.4222	34.5079	2.0857	1.0409	2.0038	0.047646	0.391097	0.388323	small	*
B2	High	45	62	38.1333	38.1129	-0.0204	1.0316	-0.0198	0.984237	-0.003879	-0.003851	negligible	

Table S15. Summary Statistics for Common Assessments by Class Subdivided by Gender and Race/Ethnicity Subgroups

Subgroup	Identity	Assessment	Class	*n*	Mean	SD	SE	Median	Min	Max	Skewness	Kurtosis
Gender	Man	Foundations Quiz	A1	36	20.4797	3.2444	0.5407	21.085	12.42	25	-0.8765	0.2872
Gender	Man	Foundations Quiz	A2	43	20.0549	2.5622	0.3907	20.5	15.67	24.25	-0.1483	-1.1958
Gender	Man	Foundations Quiz	B1	44	20.0323	2.7779	0.4188	20.5	12.67	24.25	-0.8392	0.0173
Gender	Man	Foundations Quiz	B2	45	19.4976	3.2092	0.4784	20.52	7.2	23.27	-1.4825	2.7571
Gender	Woman	Foundations Quiz	A1	84	18.3295	4.2988	0.4690	19.46	5.75	24.75	-0.8366	0.1740
Gender	Woman	Foundations Quiz	A2	86	19.2738	3.1298	0.3375	19.54	9.67	24.25	-0.7475	0.3652
Gender	Woman	Foundations Quiz	B1	114	18.5848	3.5318	0.3308	19.085	7.25	24.75	-0.8453	0.5192
Gender	Woman	Foundations Quiz	B2	125	18.9121	3.7517	0.3356	19.6	5.7	24.1	-0.8741	0.4230
Race/Ethnicity	Not White Only	Foundations Quiz	A1	33	16.9652	4.8572	0.8455	18	5.75	24.17	-0.3894	-0.9140
Race/Ethnicity	Not White Only	Foundations Quiz	A2	35	18.9143	3.5542	0.6008	19.33	9.67	24	-0.8706	0.2803
Race/Ethnicity	Not White Only	Foundations Quiz	B1	61	18.3911	3.9924	0.5112	19.67	7.25	23.75	-1.0032	0.2551
Race/Ethnicity	Not White Only	Foundations Quiz	B2	67	19.0076	3.2636	0.3987	19.6	10.6	24.1	-0.5351	-0.5294
Race/Ethnicity	White Only	Foundations Quiz	A1	91	19.6843	3.4799	0.3648	20	6	25	-1.0279	1.4929
Race/Ethnicity	White Only	Foundations Quiz	A2	94	19.7366	2.6840	0.2768	19.875	13	24.25	-0.3108	-0.7164
Race/Ethnicity	White Only	Foundations Quiz	B1	94	19.2603	2.9125	0.3004	19.5	12.67	24.75	-0.3799	-0.7096
Race/Ethnicity	White Only	Foundations Quiz	B2	102	19.0475	3.8778	0.3840	20.05	5.7	24.1	-1.1303	1.0592
Gender	Man	ACS Exam	A1	36	35.6111	7.2910	1.2152	35	24	50	0.2756	-0.9074
Gender	Man	ACS Exam	A2	43	37.6512	5.9717	0.9107	38	25	48	-0.0989	-0.7826
Gender	Man	ACS Exam	B1	44	36.2500	6.9320	1.0450	38	20	46	-0.7113	-0.1263
Gender	Man	ACS Exam	B2	45	34.9556	5.7444	0.8563	35	15	49	-0.5881	2.1673
Gender	Woman	ACS Exam	A1	84	30.9405	7.4370	0.8114	31	17	43	-0.2508	-1.1173
Gender	Woman	ACS Exam	A2	86	33.5581	7.2851	0.7856	34	17	47	-0.3474	-0.4439
Gender	Woman	ACS Exam	B1	114	33.3947	5.8893	0.5516	34	20	45	-0.2139	-0.6592
Gender	Woman	ACS Exam	B2	125	34.3680	6.2933	0.5629	34	19	47	-0.1273	-0.6742
Race/Ethnicity	Not White Only	ACS Exam	A1	33	29.1212	7.9205	1.3788	29	17	48	0.3341	-0.6678
Race/Ethnicity	Not White Only	ACS Exam	A2	35	35.2000	6.5968	1.1151	35	19	46	-0.2692	-0.7333
Race/Ethnicity	Not White Only	ACS Exam	B1	61	33.8361	6.9021	0.8837	34	20	46	-0.1718	-0.8005
Race/Ethnicity	Not White Only	ACS Exam	B2	67	34.2537	5.6282	0.6876	34	22	47	0.0394	-0.7295
Race/Ethnicity	White Only	ACS Exam	A1	91	33.4725	7.2286	0.7578	35	17	50	-0.1816	-0.5497
Race/Ethnicity	White Only	ACS Exam	A2	94	34.6702	7.2206	0.7448	34.5	17	48	-0.4113	-0.1391
Race/Ethnicity	White Only	ACS Exam	B1	94	34.3085	6.0054	0.6194	35	21	46	-0.3165	-0.5280
Race/Ethnicity	White Only	ACS Exam	B2	102	34.5882	6.4874	0.6423	34	15	49	-0.3166	0.1119

Table S16. Mean ACS Exam Scores Adjusted for Foundations Quiz Scores by Class Subdivided by Gender and Race/Ethnicity Subgroups

Subgroup	Identity	Class	*n*	Adjusted Mean	Adjusted SE	*df*	*t*-ratio
Gender	Man	A1	36	34.1146	0.9223	568	36.9870
Gender	Man	A2	43	36.5988	0.8421	568	43.4605
Gender	Man	B1	44	35.2213	0.8324	568	42.3111
Gender	Man	B2	45	34.4858	0.8212	568	41.9966
Gender	Woman	A1	84	31.6918	0.6025	568	52.6000
Gender	Woman	A2	86	33.3223	0.5938	568	56.1174
Gender	Woman	B1	114	33.8792	0.5165	568	65.5953
Gender	Woman	B2	125	34.5103	0.4925	568	70.0781
Race/Ethnicity	Not White Only	A1	33	31.3427	0.9827	568	31.8956
Race/Ethnicity	Not White Only	A2	35	35.3429	0.9445	568	37.4203
Race/Ethnicity	Not White Only	B1	61	34.5368	0.7168	568	48.1850
Race/Ethnicity	Not White Only	B2	67	34.2971	0.6826	568	50.2437
Race/Ethnicity	White Only	A1	91	32.7942	0.5873	568	55.8413
Race/Ethnicity	White Only	A2	94	33.9361	0.5781	568	58.6979
Race/Ethnicity	White Only	B1	94	34.0823	0.5765	568	59.1223
Race/Ethnicity	White Only	B2	102	34.5891	0.5532	568	62.5216

Table S17. ANCOVA Type 2 for Foundations Quiz-Adjusted ACS Exam Scores Among Classes Subdivided by Gender and Race/Ethnicity Subgroups

Subgroup	Identity	*n*	*df*	df_{error}	ss_{effect}	ss_{error}	Statistic	*p*-value	Partial η^2	Partial ω^2	Effect Size	Significance
Gender	Man	168	3	163	150.0155	4393.30104	1.8553	0.139259	0.033019	0.015132	small	
Gender	Woman	409	3	404	430.3725	12685.5029	4.5687	0.003679	0.032813	0.025570	small	**
Race/Ethnicity	Not White Only	196	3	191	338.6658	5721.20278	3.7687	0.011657	0.055887	0.040856	small	*
Race/Ethnicity	White Only	381	3	376	168.8437	11972.5922	1.7675	0.152897	0.013906	0.006023	negligible	

Table S18. Pairwise Comparisons of Foundations Quiz-Adjusted ACS Exam Scores Between Classes Subdivided by Gender and Race/Ethnicity Subgroups

Subgroup	$\sigma_{residual}$	$df_{residual}$	Identity	Comparison	Estimate	SE	*df*	Statistic	*p*-value	Cohen's *d*	Hedges' *g*	Effect Size	Significance
Gender	5.5049	568	Man	A1 - A2	-2.4842	1.2439	568	-1.9971	0.1903	-0.4513	-0.4507	small	
Gender	5.5049	568	Man	A1 - B1	-1.1067	1.2375	568	-0.8943	0.8078	-0.2010	-0.2008	small	
Gender	5.5049	568	Man	A1 - B2	-0.3712	1.2326	568	-0.3012	0.9905	-0.0674	-0.0673	negligible	
Gender	5.5049	568	Man	A2 - B1	1.3775	1.1805	568	1.1669	0.6480	0.2502	0.2499	small	
Gender	5.5049	568	Man	A2 - B2	2.1130	1.1745	568	1.7990	0.2749	0.3838	0.3833	small	
Gender	5.5049	568	Man	B1 - B2	0.7354	1.1676	568	0.6299	0.9224	0.1336	0.1334	negligible	
Gender	5.5049	568	Woman	A1 - A2	-1.6305	0.8468	568	-1.9255	0.2185	-0.2962	-0.2958	small	
Gender	5.5049	568	Woman	A1 - B1	-2.1874	0.7918	568	-2.7627	0.0301	-0.3973	-0.3968	small	*
Gender	5.5049	568	Woman	A1 - B2	-2.8185	0.7776	568	-3.6246	0.0018	-0.5120	-0.5113	medium	**
Gender	5.5049	568	Woman	A2 - B1	-0.5569	0.7876	568	-0.7071	0.8943	-0.1012	-0.1010	negligible	
Gender	5.5049	568	Woman	A2 - B2	-1.1880	0.7716	568	-1.5397	0.4144	-0.2158	-0.2155	small	
Gender	5.5049	568	Woman	B1 - B2	-0.6311	0.7132	568	-0.8849	0.8127	-0.1147	-0.1145	negligible	
Race/Ethnicity	5.5874	568	Not White Only	A1 - A2	-4.0002	1.3620	568	-2.9369	0.0181	-0.7159	-0.7150	medium	*
Race/Ethnicity	5.5874	568	Not White Only	A1 - B1	-3.1941	1.2112	568	-2.6372	0.0426	-0.5717	-0.5709	medium	*
Race/Ethnicity	5.5874	568	Not White Only	A1 - B2	-2.9544	1.1962	568	-2.4699	0.0658	-0.5288	-0.5281	medium	
Race/Ethnicity	5.5874	568	Not White Only	A2 - B1	0.8060	1.1853	568	0.6800	0.9047	0.1443	0.1441	negligible	
Race/Ethnicity	5.5874	568	Not White Only	A2 - B2	1.0458	1.1653	568	0.8974	0.8062	0.1872	0.1869	negligible	
Race/Ethnicity	5.5874	568	Not White Only	B1 - B2	0.2397	0.9897	568	0.2423	0.9950	0.0429	0.0429	negligible	
Race/Ethnicity	5.5874	568	White Only	A1 - A2	-1.1419	0.8217	568	-1.3897	0.5063	-0.2044	-0.2041	small	
Race/Ethnicity	5.5874	568	White Only	A1 - B1	-1.2881	0.8222	568	-1.5667	0.3986	-0.2305	-0.2302	small	
Race/Ethnicity	5.5874	568	White Only	A1 - B2	-1.7948	0.8068	568	-2.2246	0.1178	-0.3212	-0.3208	small	
Race/Ethnicity	5.5874	568	White Only	A2 - B1	-0.1462	0.8156	568	-0.1793	0.9980	-0.0262	-0.0261	negligible	
Race/Ethnicity	5.5874	568	White Only	A2 - B2	-0.6529	0.8002	568	-0.8160	0.8469	-0.1169	-0.1167	negligible	
Race/Ethnicity	5.5874	568	White Only	B1 - B2	-0.5067	0.7990	568	-0.6342	0.9210	-0.0907	-0.0906	negligible	

Table S19. Within-Class Gender and Race/Ethnicity Subgroup Contrasts of Foundations Quiz-Adjusted ACS Exam Scores (Type 3)

Subgroup	Model	Term	*df*	df_{error}	ss_{effect}	ss_{error}	Statistic	*p*-value	Partial η^2	Partial ω^2	Effect Size	Sig.
Gender	ACS ~ Class * Gender + Foundations	Class	3	568	259.7128	17212.6453	2.8568	0.036506	0.014864	0.009644	negligible	*
Gender	ACS ~ Class * Gender + Foundations	Gender	1	568	352.1060	17212.6453	11.6191	0.000699	0.020046	0.018289	small	***
Gender	ACS ~ Class * Gender + Foundations	Foundations	1	568	7596.0593	17212.6453	250.6623	0.000000	0.306185	0.304592	large	***
Gender	ACS ~ Class * Gender + Foundations	Class:Gender	3	568	186.5353	17212.6453	2.0518	0.105558	0.010721	0.005486	negligible	
Gender	ACS ~ Class * Gender + Foundations	Residuals	568	568	17212.6453	17212.6453	NA	NA	0.500000	0.000000	negligible	
Race/Ethnicity	ACS ~ Class * Race + Foundations	Class	3	568	446.1703	17732.3615	4.7639	0.002737	0.024544	0.019358	small	**
Race/Ethnicity	ACS ~ Class * Race + Foundations	Race	1	568	0.1024	17732.3615	0.0033	0.954356	0.000006	0.000000	negligible	
Race/Ethnicity	ACS ~ Class * Race + Foundations	Foundations	1	568	7860.0178	17732.3615	251.7708	0.000000	0.307123	0.305531	large	***
Race/Ethnicity	ACS ~ Class * Race + Foundations	Class:Race	3	568	111.8579	17732.3615	1.1943	0.311177	0.006269	0.001018	negligible	
Race/Ethnicity	ACS ~ Class * Race + Foundations	Residuals	568	568	17732.3615	17732.3615	NA	NA	0.500000	0.000000	negligible	
