# Supplementary material for: Learning Gains in Organic Chemistry from Full and Hybrid Specifications Grading
Source: JACS Au. 2026 Sep 3;6(9):4956–67. doi: 10.1021/jacsau.6c00531 (PMC13625744; doi:10.1021/jacsau.6c00531)
Supplement: Supplementary file 2 [file au6c00531_si_002.pdf]

Chem 210: Organic Chemistry I, Section 1 — Fall 2024

Welcome to Chem 210 – I am very excited to have you in this course! **Please read and understand this syllabus.** It outlines the resources and guidelines designed to best help you learn. Everything is accessible from Canvas. Above all, your, TAs, LAs, and I are here to help you learn and have a positive experience!

Class Sessions: MWF 10:10–11:00 am, [redacted]

Course Instructor: Dr. Eric Nacsa [redacted]
Preferred salutation: "Eric," Pronouns: he/him

Teaching Assistants: [redacted]

Learning Assistants:

Student Hours: About 12 hours per week – see our Canvas page for up-to-date times

Required Materials:

- *Organic Chemistry*, by Brown, Iverson, Anslyn, & Foote, 8th Ed. with access to OWLv2 (links to purchase and access in Canvas 'Modules' tab);
  TAs have physical copies of Organic Chemistry Student Solutions Manual, use the Piazza channel to arrange a time to see them
- **Approximately 25 Worksheets**: these will be the class your notes;
  new worksheets become available on Canvas by noon the day before classes that need them – download to your tablet, print yourself, or request printed copies to pick up in class all semester by adding yourself to the 'Paper Copies of Worksheets' group in the Canvas 'People' tab
- Molecular model kit (cheapest from [redacted] but any kit will do)
- Top Hat (linked through Canvas): join code [redacted] if not enrolled
- Gradescope (linked through Canvas): join code [redacted] if not enrolled

Important Dates:

- September 2 (Monday of week 2): we begin recording credit for in-class polls, team uploads, and recitations with Top Hat and Gradescope
  you need access to these apps – ensure you do and use the join codes if not
- August 31 at 11:59 pm is the deadline to drop the course without a record
- November 15 at 11:59 pm is the last day to late drop the course with a "WN"
- A complete course schedule can be found on Canvas

Biggest Navigation Tip:

- **Canvas!!** All logistic information, notes, resources, and links to other apps can be found on our Canvas page. If ever in doubt, look here!

COURSE OBJECTIVES

Students will learn concepts and principles of organic chemistry: nomenclature of organic compounds, the relationship between structure and reactivity, the reactivities of saturated and unsaturated hydrocarbons (alkanes, alkenes, alkynes) as well as alkyl halides and alcohols, and organic spectroscopy. A second goal is to learn the importance of organic chemistry and appreciate its impact on everyday life!

Learning Objectives. *After completing this course, you will be able to…*

- predict reactivity and function based on molecular structure (identifying acidic/basic and electrophilic/nucleophilic sites within compounds).
- draw and explain mechanisms of organic reactions (addition, substitution, and elimination).
- leverage knowledge of individual reactions to multistep synthesis.
- use data such from experiments such as mass spectrometry, IR spectroscopy, and NMR spectroscopy to determine the structure of organic compounds.
- make connections of organic chemistry to everyday life (biological processes, food chemistry, pharmaceuticals).

STRATEGIES FOR SUCCESS

Few students have had prior exposure to organic chemistry, so your biggest keys to success are an interest in learning new concepts, engaging with the resources provided to you, and the willingness to make mistakes and learn from them. We are committed to guiding you through this practice on your way to mastering the materials. Here are a few strategies to succeed:

1. Please come to classes and recitations with the required materials and **actively participate**. You should read assigned textbook sections before class and work the in-text and OWLv2 problems after class. This approach will significantly improve your comprehension of the subject matter.
2. **Ask questions!** In class, in recitation, at student hours, on Piazza... Students are not expected to understand everything the first (or second, or third...) time. Take advantage of student hours and review sessions. Seriously, we want to help you!!!
3. Review your class notes, compare them with the book, and make additional notes to clarify the subject matter. Complete the first and last pages of in-class worksheets on your own to define key terms and wrap everything together, respectively.
4. **Practice!** Organic chemistry is a new language! You learn a new language by using it, not by reading about it and understanding. Beyond the in-text and OWLv2 problems, the Canvas "study resources" tab also has several further topic-specific problem sets and practice exams. Complete them without your notes or the answers in front of you. You don't learn by seeing the answer and saying 'oh, I can do that.' Similarly, *only take the practice exam once you feel ready for the real exam and take it under 'real' conditions – 50 minutes, no notes!*
5. **Work in Groups!** The best way to learn something, or to find out what you don't really understand, is to try and teach it to someone else. (Trust me – I have learned more teaching than I did in the classroom as a student!)

You should spend 6–9 hours each week studying (reading, doing homework, working on practice problems, etc.) for this course. You cannot afford to fall behind. For details on University expectations, please visit: [redacted]

GRADING

**Letter Grade**	F	D	C	C+	B-	B	B+	A-	A
**Percentage**	0	57	69	77	80	83	87	90	93

A student whose overall percent grade is 83 or more and below 87 would earn a B. If everyone has a final grade in the A range, then everyone will receive an A. My goal is for you to succeed in our class!

Percentage Breakdown

Summative Assessments	Organic Foundations (Canvas & [redacted]	5%
	Midterm Exams (Tuesdays 10/1, 10/22, 11/12, & 12/10, 8–9 pm)	40%
	Final Exam	25%
Formative Assessments	Homework (OWLv2, linked through Canvas, due Sundays)	10%
Participation	In-Class Team Uploads (Gradescope, linked through Canvas)	10%
Participation	In-Class Polls (Top Hat, linked through Canvas)	5%
Participation	Recitation Participation (Top Hat, linked through Canvas)	5%
	**Total**	**100%**

Organic Foundations (5%)
This Canvas module reviews the general chemistry topics most relevant to organic chemistry. We will leverage them all semester. There is a series of videos and five Canvas practice quizzes (unlimited retakes!) Your average practice quiz score will account for 50% of your Organic Foundations grade. The second half of your score will come from a 40-minute, multiple-choice quiz administered in [redacted] on September 5–6. You must sign up for a time slot on the Penn State e-testing website at [redacted] Scheduling begins August 29 (first-come, first-serve).

Midterm Exams (40%)
Each of the 4 major units of the course will have a cumulative midterm exam focusing on the most recent unit. Exams are on Tuesdays (10/1, 10/22, 11/12, and 12/10) at 8:00–9:00 pm in [redacted] If you use all the materials provided and follow **all** the recommendations on this syllabus Worksheet 0, you will be well-prepared. There are no 'trick questions' in this course. The only items you can use are your pencil, scratch paper, and molecular model kit. A reference sheet of key terms and data will be provided – this course is *not* about memorization! To accommodate a missed exam or unforeseen challenge, everyone's lowest midterm grade will be replaced automatically with their second lowest.

Final Exam (25%)
A cumulative final multiple-choice exam will be held during finals week. Please do not make end-of-semester travel plans until after the final exam schedule is published by the Registrar, typically in October.

Homework (10%) – ***done online by each Sunday night***
Short online problem sets are due once per week (Sundays at 11:55 pm) to give you low-stakes practice and immediate feedback. Each OWLv2 problem set is accessed through a Canvas assignment. You have up to 10 attempts for each question! There will be 15 assignments, and your lowest 3 scores will be automatically dropped. During sign up and throughout the term, if you have any technical problems or grading issues, please contact Cengage Support (via the headset icon in the upper right of the OWL page). The teaching team has no ability to address technical issues and will direct you there anyway.

Participation (20%) – *all completed and submitted in-class*
Mastering any complex topic requires active engagement and grappling with the material while learning it. Therefore, our class sessions will combine 'traditional' lecture, guided sample problems, polling the class with short multiple-choice questions, and solving problems in small groups. Small, informal weekly recitations also emphasize group work while making it easy to ask questions. Among these activities, the 'participation' work, where you submit answers in-class, will help you learn and provide vital feedback to me about how well the class is following along so I can adjust instruction in real time.

Items to bring to class *every day*:

- a pen or pencil OR a tablet to complete worksheets (see page 1 for worksheet details)
- a device for answering Top Hat polls and submitting in-class team uploads to Gradescope *(all third-party apps are accessed through Canvas – see page 1 for details)*

In-Class Polls (5%): I will frequently ask the class short questions to gauge your understanding of the material we are working on. Your responses will be analyzed in Top Hat to provide a breakdown of the class distribution in real time and will only be graded for participation. You earn full credit in this category for the semester by answering 80% of polls (no bonus credit accrues beyond 80%).

In-Class Team Uploads (10%): About once per week, teams of up to 4 students will upload a photo or screenshot of their work from a short activity using Gradescope. Your submission ***must*** include in-class work only, so the photo or screenshot must be taken by 11:05 am. You are *encouraged* to upload your work before leaving class. To avoid unnecessary pressure and avoid tech issues, responses will be accepted until midnight, but images uploaded made after 11:05 am ***must*** include a time stamp showing they were taken at 11:05 am or earlier. Only one submission per team is needed. The student making the upload needs to add all teammates to the Gradescope submission so everyone gets credit. Since mistakes happen, however, all images uploaded must also contain clearly written names of all teammates. This safeguard ensures that a student who was not included in the Gradescope team in error has evidence they participated, and it is each student's responsibility to verify that the image includes their name. Use your notes and ask questions of the TAs, LAs, and your peers! **We are here to help!** You earn full credit in this category for the semester by earning 80% of the possible points (no bonus credit accrues beyond 80%). Furthermore, any good-faith attempt uploaded on time will earn 40%, no matter how incorrect.

Recitations (5%): These weekly sessions are a great chance to reinforce new concepts in a comfortable setting, with familiar faces, and where getting extensive feedback from your TA is a lot easier than in class. Recitations have their own worksheets that will be available on Canvas by the end of each Monday. Come with a pen/pencil or tablet, a device to sign in on Top Hat, and be ready to work with your classmates! Recitations account for 5% of your grade. Attendance is all that matters. There is no penalty for making mistakes! You earn full credit for the semester by attending 12 of the 14 recitations.

Extra credit (4+%)
There is ample opportunity for extra credit!

Names from a Hat (1+%): On the first day of class, anyone may earn 0.5% extra credit by putting a notecard with your name in hat. At times during the semester, I will draw names from the hat after I ask a question to the class. If your name is drawn and you answer, you earn another 0.5% (whether or not the answer is correct!) If you change your mind partway through the semester and have your name either added or removed from the hat, just ask. Please only opt in if you plan to attend (almost) every class and intend to answer whenever called on. It wastes everyone's time if I read a bunch of names and no one replies. For this reason, if you are called on and do not answer, you forfeit the initial 0.5% extra credit and are removed for the rest of the semester (no net penalty). **If you answer** and earn the second 0.5% extra credit, your name will be put back so **you can continue earning more bonus points!**

Drug Prices Case Study (1%): At the start of unit 2, we will analyze ethical issues surrounding the price of medicines in the US. You can earn up to 1% extra credit by completing these activities.

Asking Questions on Piazza (1%): If you use Piazza (see below) as your only form of electronic communication with the teaching team for the whole semester, you will earn this extra credit.

Answering Questions on Piazza (1%): Questions asked on Piazza (see below) are seen by the whole class (although only your instructor and the TA see your identity) and can be answered by anyone in the whole class. Help your classmates get answers faster by replying to them! The 20 students with the most initial (and correct) answers to classmates' questions through the semester will earn this credit.

What if I miss a class, recitation, assignment, or exam?
Every assessment category has generous forgiveness policies as described earlier. The reason for this flexibility is to avoid asking you to justify your absence and having us approve or reject subjective situations. If you miss any of these items for any reason, no matter how trivial or serious, please do not worry about explaining yourself. The missed assessment will be dropped per course policies, and thus will not lower your grade. If you missed so many opportunities in single category for a reason beyond your control that those absences made you unable to earn full credit (i.e., you missed 2+ exams, 4+ homework assignments, over 20% of the in-class polls for the whole semester, over 20% of the in-class team upload points for the whole semester, or 3+ recitations), please reach out and we will develop a plan to make up the missed work.

Regrades & Grade Corrections
If disagree with the grading on any exam or other graded item, you are invited to submit a regrade request. All re-grades must be made in-person and with everything on paper within a week of the exam/assignment's return. Submit (1) your entire exam/assignment, (2) the key, and (3) a detailed explanation justifying your request. Items (1) and (2) must be clearly annotated in a new color to draw attention to the relevant items. Comparisons to other students' exams will not be considered. Along with your specific request, your entire exam/assignment will be regraded.

Deferred grades
Deferred grades are granted only in special circumstances when, for reasons beyond a student's control, a student is verifiably prevented from completing a course within the normal time. It is the student's obligation to request the deferral and take a replacement final exam before the university-set deadlines.

FURTHER RESOURCES & COMMUNICATION

Student Hours – ***the best way to ask questions to your instructor, TAs, and LAs outside of class***
Many simple questions are best answered before or after class. If you have anything more complex or cannot get to us in time, we would love to see you in student hours! Please drop in for clarifications, logistic questions, study tips, or just to chat! (See Canvas for times). If the wait for student hours is too long, consider Piazza (the fastest electronic option, see below). Canvas messages will not be answered. I will answer your emails, but although bonus credit will forfeited.

Canvas – ***good for everything (except private contact with your instructor, TAs, and LAs)***
Canvas has everything you need! The current and upcoming worksheets, past notes, reading, textbook problems, and extra practice problems are on the main page. Links to all electronic resources and apps (e-text, OWLv2, Top Hat, Gradescope) are in tabs. All assignments beyond in-class activities and quizzes are hosted or linked through Canvas, including a due date to keep you ready and a grade so you can track your progress. I will also use the announcements feature to communicate the whole class outside of our lecture times. Check these regularly and ensure you get email notifications, but do not contact us directly with Canvas messages. They are simply too cumbersome to answer, so do not expect a response. Please ask questions in-person or on Piazza (or by email if you must).

Piazza – ***the best means of (electronic) communication and asking (non-personal) questions***
This amazing class discussion forum and is integrated into Canvas. Anything you post can be seen by the whole class, although your identity is only seen by the instructor and TAs. Please ask your questions here unless wide distribution is inappropriate. Why? Piazza is the fastest way to get your question answered since the whole class can respond! Also, teaching others improves your own understanding of a topic, and I am always impressed by the quality of peer answers! You earn 1% extra credit by using Piazza as your only form of electronic communication with the teaching team throughout the semester, and another 1% extra credit will be awarded to the 10 students with the most initial (correct) answers to classmates' questions throughout the semester!

Email – ***please only use if necessary***
It has its place, but it is not a good way to answer questions in a large class. With so many of us, students often have similar questions, but with email, only the sender learns the answer. Please use email only if a message is not appropriate to post to Piazza and if you cannot reach me in-person (or electronic records are needed). Routine emails about course content, logistics, etc., will be answered within 48 M–F hours, but you will forfeit the extra credit from sticking to Piazza for electronic communication all semester, and the response will be slower than Piazza. Messages that should clearly not be broadcast on Piazza and need electronic records (SDR paperwork, requests to meet outside of class/student hours, personal emergencies, etc.) will be answered promptly and will not forfeit Piazza extra credit. Requests that ignore course policies (requests for any regrades before finals week, extensions on a single assignment, etc.) might not be answered at all, so please read this syllabus thoroughly!

UNIVERSITY POLICIES OF NOTE

Counseling and Psychological Services (CAPS)

Many students at Penn State face personal challenges or have psychological needs that may interfere with interfere with their academic progress, social development, or emotional wellbeing. The university offers a variety of confidential services to help you through difficult times, including individual and group counseling, crisis intervention, consultations, online chats, and mental health screenings. These services are provided by staff who welcome all students and embrace a philosophy respectful of clients' cultural and religious backgrounds, and sensitive to differences in race, ability, gender identity and sexual orientation.

[redacted]

Student Disability Resources (SDR)

Penn State welcomes students with disabilities into the University's educational programs. *If you have academic accommodations, please see me in my office for testing arrangements ASAP.* For further information, please visit Student Disability Resources Web site: [redacted] Their educational counselors can help with time management, reading, math learning skills, note-taking and exam preparation skills. All their services are *free* to PSU students.

In order to receive consideration for reasonable accommodations, you must contact SDR, participate in an intake interview, and provide documentation: [redacted] the documentation supports your request for reasonable accommodations, your campus's disability services office will provide you with an accommodation letter. Please share this letter with your instructors and discuss the accommodations with them as early in your courses as possible. You must follow this process for every semester that you request accommodations.

Educational Equity/Report Bias

Penn State takes great pride to foster a diverse and inclusive environment for students, faculty, and staff. Acts of intolerance, discrimination, or harassment due to age, ancestry, color, disability, gender, gender identity, national origin, race, religious belief, sexual orientation, or veteran status are not tolerated and can be reported through Educational Equity via [redacted]

Honor Code Issues and Academic Integrity

You are reminded of the Academic Integrity Policy in the University Code of Conduct: "Academic integrity is the pursuit of scholarly activity in an open, honest and responsible manner. Academic integrity is a basic guiding principle for all academic activity at The Pennsylvania State University, and all members of the University community are expected to act in accordance with this principle. Consistent with this expectation, the University's Code of Conduct states that all students should act with personal integrity, respect other students' dignity, rights and property, and help create and maintain an environment in which all can succeed through the fruits of their efforts.

Academic integrity includes a commitment by all members of the University community *not to engage in or tolerate acts of falsification, misrepresentation or deception.* Such acts of dishonesty violate the fundamental ethical principles of the University community and compromise the worth of work completed by others."

For more information on Academic Integrity, please see the Undergraduate Advising Handbook: [redacted]

All Penn State [redacted] and Eberly College of Science [redacted] academic integrity policies and procedures regarding ethics and honorable behavior apply to this course. Academic dishonesty includes, but is not limited to, cheating, plagiarizing, fabrication of information or citations, facilitating acts of academic dishonesty by others, having unauthorized possession of examinations or other unauthorized class materials, submitting work of another person or work previously used without informing the instructor, or tampering with the academic work of other students. In a broader context, you should be familiar with and follow the Penn State Code of Conduct and the Code of Mutual Respect and Cooperation.

Specific instances of academic dishonesty in this course include, but are not limited to:

- Representing yourself to be another person online
- Allowing another person to represent you online
- Allowing unauthorized persons to access lecture materials, quizzes, or exams
- Copying or helping someone else copy during an examination
- Receiving help or information from any person during a quiz or exam (this includes from websites such as Chegg and similar)
- Using unauthorized materials or notes during quizzes or examinations
- Searching for quiz or test answers on the internet
- Using a text-programmable calculator on quizzes or examinations
- Using a cell phone or other communication device during a quiz or exam
- Stealing or destroying course materials
- Sharing information about quiz and exam questions with other students
- Altering answers or grades on graded examinations
- Having notes or extra papers of any kind out during an exam
- Having someone take an examination for you
- Keeping materials from a conflict or make-up exam
- Distributing course materials to others
- Providing a false excuse for missed exams
- Attempting to do any of the above

Consequences of such infractions may range from awarding a grade of "0" on the exam or assignment in question to receiving an immediate F in the course and not allowing the student to drop the class.

If you have read this far, good job. Send a funny photo of an animal to [redacted] by the end of the semester. The funniest one will earn one percent extra credit at the end of the semester. Note that I do not check this account until finals week, and if you tell other students about this chance, you are less likely to win.

We are here to help you learn and succeed! We will listen if you have any concern or something comes up. It is always better to be honest and talk to us than to resort to academic dishonesty. We thank you in advance for following these guidelines.
