# Supplementary material for: Learning Gains in Organic Chemistry from Full and Hybrid Specifications Grading
Source: JACS Au. 2026 Sep 3;6(9):4956–67. doi: 10.1021/jacsau.6c00531 (PMC13625744; doi:10.1021/jacsau.6c00531)
Supplement: Supplementary file 3 [file au6c00531_si_003.pdf]

Welcome to Chem 210 – I am very excited to have you in this course! **Please read and understand this syllabus.** It outlines the resources and guidelines designed to best help you learn. Everything is accessible from Canvas. Above all, your, TAs, LAs, and I are here to help you learn and have a positive experience!

Class Sessions: MWF 11:15 am – 12:05 pm, [redacted]

Course Instructor: Dr. Eric Nacsa[redacted]
Preferred salutation: "Eric," Pronouns: he/him

[redacted]

Teaching Assistants:

Learning Assistants:

GSG Leader:

Student Hours: About 12 hours per week – see our Canvas page for up-to-date times

Required Materials:

- *Organic Chemistry*, by Brown, Iverson, Anslyn, & Foote, 8th Ed. with access to OWLv2 (links to purchase and access in Canvas 'Modules' tab);
  TAs have physical copies of Organic Chemistry Student Solutions Manual, use the Piazza channel to arrange a time to see them
- **25 Worksheets**: these will be the class your notes;
  new worksheets become available on Canvas by noon the day before classes that need them – download to your tablet, print yourself, or request printed copies to pick up in class all semester by adding yourself to the 'Paper Copies of Worksheets' group in the Canvas 'People' tab
- Molecular model kit (cheapest from [redacted] but any kit will do)
- Top Hat (linked through Canvas): join code [redacted] if not enrolled
- Gradescope (linked through Canvas): join code [redacted] if not enrolled

Important Dates:

- August 31 at 11:59 pm is the deadline to drop the course without a record
- Wednesday September 3: we begin recording credit for in-class polls, team uploads, and recitations with Top Hat and Gradescope
  you need access to these apps – ensure you do and use the join codes if not
- Thu/Fri September 4/5: Foundations Quiz at [redacted]
- Thursday September 11: Weekly Quizzes begin
- November 14 at 11:59 pm is the last day to late drop the course with a "WN"
- A complete course schedule can be found on Canvas

Biggest Navigation Tip:

- **Canvas!!** All logistic information, notes, resources, and links to other apps can be found on our Canvas page. If ever in doubt, look here!

COURSE OBJECTIVES

Students will learn concepts and principles of organic chemistry: nomenclature of organic compounds, the relationship between structure and reactivity, the reactivities of saturated and unsaturated hydrocarbons (alkanes, alkenes, alkynes) as well as alkyl halides and alcohols, and organic spectroscopy. A second goal is to learn the importance of organic chemistry and appreciate its impact on everyday life!

Learning Objectives. *After completing this course, you will be able to…*

- predict reactivity and function based on molecular structure (identifying acidic/basic and electrophilic/nucleophilic sites within compounds).
- draw and explain mechanisms of organic reactions (addition, substitution, and elimination).
- leverage knowledge of individual reactions to multistep synthesis.
- use data such from experiments such as mass spectrometry, IR spectroscopy, and NMR spectroscopy to determine the structure of organic compounds.
- make connections of organic chemistry to everyday life (biological processes, food chemistry, pharmaceuticals).

STRATEGIES FOR SUCCESS

Few students have had prior exposure to organic chemistry, so your biggest keys to success are an interest in learning new concepts, engaging with the resources provided to you, and the willingness to make mistakes and learn from them. We are committed to guiding you through this practice on your way to mastering the materials. Here are a few strategies to succeed:

1. Please come to classes and recitations with the required materials and **actively participate**. You should read assigned textbook sections before class and work the in-text and OWLv2 problems after class. This approach will significantly improve your comprehension of the subject matter.
2. **Ask questions!** In class, in recitation, at student hours, on Piazza... Students are not expected to understand everything the first (or second, or third...) time. Take advantage of student hours and review sessions. Seriously, we want to help you!!!
3. Review your class notes, compare them with the book, and make additional notes to clarify the subject matter. Complete the first and last pages of in-class worksheets on your own to define key terms and wrap everything together, respectively.
4. **Practice!** Organic chemistry is a new language! You learn a new language by using it, not by reading about it and understanding. Beyond the in-text and OWLv2 problems, the Canvas "study resources" tab also has several further topic-specific problem sets. Complete them without your notes or the answers in front of you. You don't learn by seeing the answer and saying 'oh, I can do that.'
5. **Work in Groups!** The best way to learn something, or to find out what you don't really understand, is to try and teach it to someone else. (Trust me – I have learned more teaching than I did in the classroom as a student!)

You should spend 6–9 hours each week studying (reading, doing homework, working on practice problems, etc.) for this course. You cannot afford to fall behind. For details on University expectations, please visit: [redacted]

GRADING

**Letter Grade**	F	D	C	C+	B-	B	B+	A-	A
**Percentage**	0	57	67	77	80	83	87	90	93

A student whose overall percent grade is 83 or more and below 87 would earn a B. If everyone has a final grade in the A range, then everyone will receive an A. My goal is for you to succeed in our class!

Percentage Breakdown

Summative Assessments	Learning Target Quizzes	80%
	Final Exam	10%
Formative Assessments	Homework (OWLv2, linked through Canvas, due Sundays)	2%
Participation	In-Class Polls (Top Hat, linked through Canvas)	2%
	In-Class Team Uploads (Gradescope, linked through Canvas)	3%
	Learning Community Participation (Top Hat, linked via Canvas)	3%
	**Total**	**100%**

Learning Target Quizzes (80%)

The grading structure of this course has been designed to reflect how much you have learned throughout the course. To reward your progress in learning from mistakes, avoid stressful, high-stakes exams, and avoid overrepresentation of certain topics, most of your grade is earned through short, highly targeted quizzes that address specific learning targets (LTs). Quizzes for each target can be taken up to four times, with only your top 1–2 outcomes counting toward your final grade. Overall, 33 quizzes will be considered, mostly weighted at 2%, although the second-highest grade from six in-semester 'Essential LTs' will count for 3%.

The first quiz, a Canvas module called the Organic Foundations Quiz, reviews the general chemistry topics most relevant to organic chemistry. We will leverage them all semester. There is a series of videos and five Canvas practice quizzes (unlimited retakes!) You must earn 4/5 points on each quiz before you can take the Organic Foundations Quiz, which is the only item in this unit that contributes to your final grade. It is a 40-minute, multiple-choice quiz administered in [redacted] on September 4–5. You must sign up for a time slot on the [redacted]

Thereafter, i.e., beginning Thursday September 11 in Week 3, LT quizzes will be offered during your Thursday recitation (9:05–10:20 am) in [redacted] with additional times becoming available at a selection of 'Quiz Hours' on Tuesdays. Details about times, locations, and sign-up procedures are TBD.

- Grades will be assigned to LT quizzes using an *EGRI* rubric.

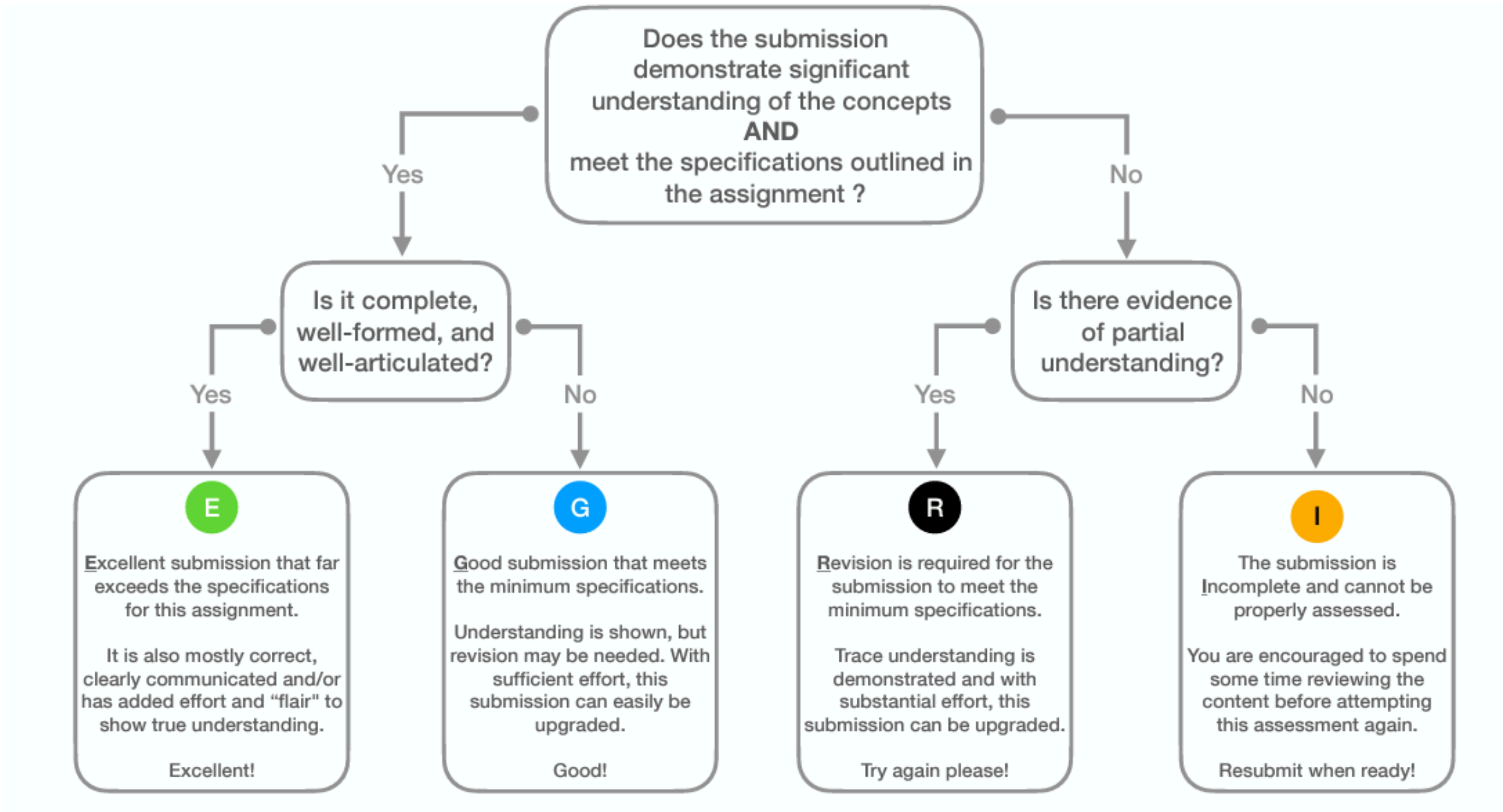

E = 2.0 points G = 1.8 points R = 0 points I = 0 points

- Participation in the Learning Target Quizzes each week is **critical**. Most learning targets will be available **four** times throughout the semester according to a schedule. Missing 1–2 quiz sessions throughout the semester is not likely to greatly impact your final grade. However, every time you miss a quiz session you miss an opportunity to improve your best quiz scores.

- **Retaking a Learning Target Quiz**: If you would like to improve your quiz scores, you will have further opportunities. Retaking a quiz is highly recommended if you have an R or I counting toward your final grade, but you can also try to earn the last 10% by upgrading from a G to and E. No points can be lost by retaking quizzes since only your highest 1–2 attempts will count toward your final grade (see chart below for how many quizzes each LT contributes).

- What constitutes "*E or G*"?

  Each learning target quiz is an opportunity for you to demonstrate that you understand the concepts related to the Learning Target and that you have developed the skills associated with that target.

  A quiz will be graded *Excellent* if it exceeds the specifications for this assignment. Think of this as A-level work (does not have to be perfect!). It is mostly correct, clearly communicated and/or has added effort and "flair" to show true understanding. A *Good* submission meets the minimum specifications (e.g. B-level work). Understanding is shown, but revision may be needed. With sufficient effort, this submission can easily be upgraded.

- Is "*revision*" on a Learning Target Quiz the same as an "F"?

  Not at all! We use "revision" to help emphasize that every time you attempt a learning target quiz, you have some understanding. Your final grade in the course is based, in part, on the number of Learning Targets you successfully complete and master. What matters is that you earn a "*E or G*" grade on each target. On some targets this may take multiple attempts, but each attempt is an opportunity for you to get feedback on your misunderstandings, to focus on what

you don't yet understand about that particular target, and then to come back to that target with another attempt. This format will help you build understanding over time and strengthen your skills in every target in the course.

- Do I need to get a "*perfect score*" on a Learning Target to earn a "*satisfactory*"?

  Absolutely not! On any one learning target quiz, a "*E or G*" means that you have demonstrated proficiency and understanding in the particular skills included in that learning target. It may feel at first that this means you need to get a "100%" in everything, but this is a misconception. Both *"E or G"* on a Learning Target Quiz is very different from getting a 100% on a one-time Midterm or Final Exam. Each Learning Target Quiz assesses a small set of skills focused around one Learning Target. After each attempt, you get feedback on that attempt. It is up to you to use this feedback to focus your review of the material before making another attempt. You earn a "*E or G"* on a Learning Target by building your understanding of that target to the point where you can demonstrate a strong understanding of that target in a short quiz. If you want to compare this process to a midterm exam, you can think of it as "getting 100%" on one small part of one exam, except that you have multiple attempts on the same part of that exam.

- Do I need to complete the Learning Targets in order?

  No. You do not need to get a "*E or G*" on LT1 before trying LT2 or LT3 (for example). Some learning targets will depend on skills developed on earlier targets and the material throughout the course is highly interconnected. It is important to continue attempting multiple learning target quizzes each week to keep pace with the course and to give yourself time for multiple attempts at each learning target. This approach differs from a setting with high-stakes exams. Each quiz attempt is simultaneously an assessment opportunity and a learning opportunity.

- How many times can I take a LTQ?

  You will have the opportunity to take most LTQs up to four (4) times. Learning targets will be available for two consecutive weeks in our Thursday recitations, followed by an additional two times during Tuesday 'Quiz Hours.'

- You've mentioned the possibility of taking LTQs during quiz hours. How does this work?

  As the semester progresses, some students may find they need to complete more quizzes than they can take in a single Thursday session. We will provide students an opportunity to sign up to take LTQs during quiz hours. You are still limited to one attempt per learning target per week.

**Target**	**# of Quizzes in Final Grade**	**Description**
**LT0 *Foundations****	1 time	Atomic structure, bonding, Lewis structure, functional groups, molecular structure
**LT1* (1.5 ×)**	2 times	Convert between condensed structural formulas, Lewis structures, and bond-line structures, draw isomers
**LT2* (1.5 ×)**	2 times	Identify and explain charge stability, formal charges, resonance
**LT3**	1 time	Organic nomenclature
**LT4* (1.5 ×)**	2 times	Conformation of alkanes & cycloalkanes

**LT5* (1.5 ×)**	2 times	Cis and trans isomerism, alkene geometry, *E*/*Z*
**LT6* (1.5 ×)**	2 times	Identify chiral and prochiral centers, indicate (*R*)/(*S*) configurations, draw stereoisomers and identify relationships
**LT7* (1.5 ×)**	2 times	Use structure or pK_a to explain acid-base properties of molecules
**LT8* (1.5 ×)**	2 times	Molecular orbital theory
**LT9**	2 times	Nucleophiles & electrophiles, mechanistic steps
**LT10**	2 times	S_N2 and E2 reactions
**LT11**	2 times	S_N1 and E1 reactions
**LT12**	2 times	Electrophilic alkene addition reactions
**LT13**	1 time	Radical stability, radical substitution of alkanes
**LT14**	2 times	Organic synthesis
**LT15**	1 time	Mass spectrometry
**LT16**	1 time	IR spectroscopy
**LT17**	2 times	NMR signals, splitting, chemical shift, integration
**LT18**	2 times	MS, IR, NMR structure determination

*Denotes an 'essential' learning target – your quiz grades in each in-semester essential LT counts 1.5 × more than others (3% instead of 2%).

Final Exam (10%)
A cumulative final multiple-choice exam will be held during finals week. Please do not make end-of-semester travel plans until after the final exam schedule is published by the Registrar, typically in October.

Homework (2%) – *due online each Sunday night*
Short online problem sets are due once per week (Sundays at 11:55 pm) to give you low-stakes practice and immediate feedback. Each OWLv2 problem set is accessed through a Canvas assignment. You have up to 10 attempts for each question! There will be 15 assignments, and your lowest 3 scores will be automatically dropped. During sign up and throughout the term, if you have any technical problems or grading issues, please contact Cengage Support (via the headset icon in the upper right of the OWL page). The teaching team has no ability to address technical issues and will direct you there anyway.

Participation (8%) – *all completed and submitted in-class*
Mastering any complex topic requires active engagement and grappling with the material while learning it. Therefore, our class sessions will combine 'traditional' lecture, guided sample problems, polling the class with short multiple-choice questions, and solving problems in small groups. Small, informal weekly recitations also emphasize group work while making it easy to ask questions. Among these activities, the 'participation' work, where you submit answers in-class, will help you learn and provide vital feedback to me about how well the class is following along so I can adjust instruction in real time.

Items to bring to class *every day*:

- a pen or pencil OR a tablet to complete worksheets (see page 1 for worksheet details)
- a device for answering Top Hat polls and submitting in-class team uploads to Gradescope
  (all third-party apps are accessed through Canvas – see page 1 for details)

In-Class Polls (2%): I will frequently ask the class short questions to gauge your understanding of the material we are working on. Your responses will be analyzed in Top Hat to provide a breakdown of the class distribution in real time and will only be graded for participation. You earn full credit in this category for the semester by answering polls in 80% of classes (no bonus credit accrues beyond 80%).

In-Class Team Uploads (3%): About once per week, teams of up to 4 students will upload a photo or screenshot of their work from a short activity using Gradescope. Your submission ***must*** include in-class

work only, so the photo or screenshot must be taken by 12:10 pm. You are *encouraged* to upload your work before leaving class. To avoid unnecessary pressure and avoid tech issues, responses will be accepted until midnight, but images uploaded made after 12:10 pm ***must*** include a time stamp showing they were taken at 12:10 am or earlier. Only one submission per team is needed. The student making the upload needs to add all teammates to the Gradescope submission so everyone gets credit. Since mistakes happen, however, all images uploaded must also contain clearly written names of all teammates. This safeguard ensures that a student who was not included in the Gradescope team in error has evidence they participated, and it is each student's responsibility to verify that the image includes their name. Use your notes and ask questions of the TAs, LAs, and your peers! **We are here to help!** Any good-faith attempt uploaded on time will earn 40%, no matter how incorrect, and only the top 80% of your submissions will count toward your final grade.

Learning Communities (3%): These weekly sessions are a great chance to reinforce new concepts in a comfortable setting, with familiar faces, and where getting extensive feedback from your TAs and LAs is a lot easier than in class. Learning Communities have their own worksheets that will be available on Canvas by the end of each weekend. Come with a pen/pencil or tablet, a device to sign in on Top Hat, and be ready to work with your classmates! Attendance account for 3% of your grade. Attendance is all that matters. There is no penalty for making mistakes! You earn full credit for the semester by attending 12 of the 14 recitations. Sign up occurs in the first week of class.

Extra credit (4+%)

There is ample opportunity for extra credit!

Names from a Hat (1+%): On the first day of class, anyone may earn 0.5% extra credit by putting a notecard with your name in hat. At times during the semester, I will draw names from the hat after I ask a question to the class. If your name is drawn and you answer, you earn another 0.5% (whether or not the answer is correct!) If you change your mind partway through the semester and have your name either added or removed from the hat, just ask. Please only opt in if you plan to attend (almost) every class and intend to answer whenever called on. It wastes everyone's time if I read a bunch of names and no one replies. For this reason, if you are called on and do not answer, you forfeit the initial 0.5% extra credit and are removed for the rest of the semester (no net penalty). **If you answer** and earn the second 0.5% extra credit, your name will be put back so **you can continue earning more bonus points!**

Drug Prices Case Study (1%): At the start of unit 2, we will analyze ethical issues surrounding the price of medicines in the US. You can earn up to 1% extra credit by completing these activities.

Asking Questions on Piazza (1%): If you use Piazza (see below) as your only form of electronic communication with the teaching team for the whole semester, you will earn this extra credit.

Answering Questions on Piazza (1%): Questions asked on Piazza (see below) are seen by the whole class (although only your instructor and the TA see your identity) and can be answered by anyone in the whole class. Help your classmates get answers faster by replying to them! The 20 students with the most initial (and correct) answers to classmates' questions through the semester will earn this credit.

What if I miss a class, quiz, or assignment?

Every assessment category has generous forgiveness policies as described earlier. The reason for this flexibility is to avoid asking you to justify your absence and having us approve or reject subjective situations. If you miss any of these items for any reason, no matter how trivial or serious, please do not worry about explaining yourself. The missed assessment will be dropped per course policies, and thus will not lower your grade. If you missed so many opportunities in single category for a reason beyond your control that those absences made you unable to earn full credit (i.e., you missed 3+ quizzes for a learning target, 4+ homework assignments, over 20% of classes for the whole semester, over 20% of the in-class team uploads for the whole semester, or 3+ learning communities) , please reach out and we will develop

a plan to make up the missed work.

Regrades & Grade Corrections
If disagree with the grading on any quiz or other graded item, you are invited to submit a regrade request. All re-grades are submitted in Gradescope and will be open for one week after an assignment is graded. Submit a detailed explanation justifying your request with clear references to the key and your work. Comparisons to other students' work will not be considered.

Deferred grades
Deferred grades are granted only in special circumstances when, for reasons beyond a student's control, a student is verifiably prevented from completing a course within the normal time. It is the student's obligation to request the deferral and take a replacement final exam before the university-set deadlines.

FURTHER RESOURCES & COMMUNICATION

Student Hours – *the best way to ask questions to your instructor, TAs, and LAs outside of class*
Many simple questions are best answered before or after class. If you have anything more complex or cannot get to us in time, we would love to see you in student hours! Please drop in for clarifications, logistic questions, study tips, or just to chat! (See Canvas for times). If the wait for student hours is too long, consider Piazza (the fastest electronic option, see below). Canvas messages will not be answered. I will answer your emails, but although bonus credit will forfeited.

Canvas – *good for everything (except private contact with your instructor, TAs, and LAs)*
Canvas has everything you need! The current and upcoming worksheets, past notes, reading, textbook problems, and extra practice problems are on the main page. Links to all electronic resources and apps (e-text, OWLv2, Top Hat, Gradescope) are in tabs. All assignments beyond in-class activities and quizzes are hosted or linked through Canvas, including a due date to keep you ready and a grade so you can track your progress. I will also use the announcements feature to communicate the whole class outside of our lecture times. Check these regularly and ensure you get email notifications, but do not contact us directly with Canvas messages. They are simply too cumbersome to answer, so do not expect a response. Please ask questions in-person or on Piazza (or by email if you must).

Piazza – *the best means of (electronic) communication and asking (non-personal) questions*
This amazing class discussion forum and is integrated into Canvas. Anything you post can be seen by the whole class, although your identity is only seen by the instructor and TAs. Please ask your questions here unless wide distribution is inappropriate. Why? Piazza is the fastest way to get your question answered since the whole class can respond! Also, teaching others improves your own understanding of a topic, and I am always impressed by the quality of peer answers! You earn 1% extra credit by using Piazza as your only form of electronic communication with the teaching team throughout the semester, and another 1% extra credit will be awarded to the 10 students with the most initial (correct) answers to classmates' questions throughout the semester!

Email – *please only use if necessary*
It has its place, but it is not a good way to answer questions in a large class. With so many of us, students often have similar questions, but with email, only the sender learns the answer. Please use email only if a message is not appropriate to post to Piazza and if you cannot reach me in-person (or electronic records are needed). Routine emails about course content, logistics, etc., will be answered within 48 M–F hours, but you will forfeit the extra credit from sticking to Piazza for electronic communication all semester, and the response will be slower than Piazza. Messages that should clearly not be broadcast on Piazza and need electronic records (SDR paperwork, requests to meet outside of class/student hours, personal emergencies,

etc.) will be answered promptly and will not forfeit Piazza extra credit. Requests that ignore course policies (requests for any regrades before finals week, extensions on a single assignment, etc.) might not be answered at all, so please read this syllabus thoroughly!

UNIVERSITY POLICIES OF NOTE

Counseling and Psychological Services (CAPS)

Many students at Penn State face personal challenges or have psychological needs that may interfere with interfere with their academic progress, social development, or emotional wellbeing. The university offers a variety of confidential services to help you through difficult times, including individual and group counseling, crisis intervention, consultations, online chats, and mental health screenings. These services are provided by staff who welcome all students and embrace a philosophy respectful of clients' cultural and religious backgrounds, and sensitive to differences in race, ability, gender identity and sexual orientation.

[redacted]

Student Disability Resources (SDR)

Penn State welcomes students with disabilities into the University's educational programs. *If you have academic accommodations, please see me in my office for testing arrangements ASAP.* For further information, please visit Student Disability Resources Web site: [redacted] Their educational counselors can help with time management, reading, math learning skills, note-taking and exam preparation skills. All their services are *free* to PSU students.

In order to receive consideration for reasonable accommodations, you must contact SDR, participate in an intake interview, and provide documentation: [redacted] If the documentation supports your request for reasonable accommodations, your campus's disability services office will provide you with an accommodation letter. Please share this letter with your instructors and discuss the accommodations with them as early in your courses as possible. You must follow this process for every semester that you request accommodations.

Educational Equity/Report Bias

Penn State takes great pride to foster a diverse and inclusive environment for students, faculty, and staff. Acts of intolerance, discrimination, or harassment due to age, ancestry, color, disability, gender, gender identity, national origin, race, religious belief, sexual orientation, or veteran status are not tolerated and can be reported through Educational Equity via [redacted]

Honor Code Issues and Academic Integrity

You are reminded of the Academic Integrity Policy in the University Code of Conduct: "Academic integrity is the pursuit of scholarly activity in an open, honest and responsible manner. Academic integrity is a basic guiding principle for all academic activity at The Pennsylvania State University, and all members of the University community are expected to act in accordance with this principle. Consistent with this expectation, the University's Code of Conduct states that all students should act with personal integrity, respect other students' dignity, rights and property, and help create and maintain an environment in which all can succeed through the fruits of their efforts.

Academic integrity includes a commitment by all members of the University community *not to engage in or tolerate acts of falsification, misrepresentation or deception.* Such acts of dishonesty violate the fundamental ethical principles of the University community and compromise the worth of work completed by others."

For more information on Academic Integrity, please see the Undergraduate Advising Handbook: [redacted]

All Penn State [redacted] and Eberly College of Science [redacted] academic integrity policies and procedures regarding ethics and honorable behavior apply to this course. Academic dishonesty includes, but is not limited to, cheating, plagiarizing, fabrication of information or citations, facilitating acts of academic dishonesty by others, having unauthorized possession of examinations or other unauthorized class materials, submitting work of another person or work previously used without informing the instructor, or tampering with the academic work of other students. In a broader context, you should be familiar with and follow the Penn State Code of Conduct and the Code of Mutual Respect and Cooperation.

Specific instances of academic dishonesty in this course include, but are not limited to:

- Representing yourself to be another person online
- Allowing another person to represent you online
- Allowing unauthorized persons to access lecture materials, quizzes, or exams
- Copying or helping someone else copy during an examination
- Receiving help or information from any person during a quiz or exam (this includes from websites such as Chegg and similar)
- Using unauthorized materials or notes during quizzes or examinations
- Searching for quiz or test answers on the internet
- Using a text-programmable calculator on quizzes or examinations
- Using a cell phone or other communication device during a quiz or exam
- Stealing or destroying course materials
- Sharing information about quiz and exam questions with other students
- Altering answers or grades on graded examinations
- Having notes or extra papers of any kind out during an exam
- Having someone take an examination for you
- Keeping materials from a conflict or make-up exam
- Distributing course materials to others
- Providing a false excuse for missed exams
- Attempting to do any of the above

Consequences of such infractions may range from awarding a grade of "0" on the exam or assignment in question to receiving an immediate F in the course and not allowing the student to drop the class.

If you have read this far, good job. Send a funny photo of an animal to [redacted] by the last class of the semester. The funniest one will earn one percent extra credit at the end of the semester. Note that I do not check this account until finals week, and if you tell other students about this chance, you are less likely to win.

We are here to help you learn and succeed! We will listen if you have any concern or something comes up. It is always better to be honest and talk to us than to resort to academic dishonesty. We thank you in advance for following these guidelines.
