# Supplementary material for: Learning Gains in Organic Chemistry from Full and Hybrid Specifications Grading
Source: JACS Au. 2026 Sep 3;6(9):4956–67. doi: 10.1021/jacsau.6c00531 (PMC13625744; doi:10.1021/jacsau.6c00531)
Supplement: Supplementary file 4 [file au6c00531_si_004.pdf]

CHEM 210: Organic Chemistry I — Section 003, Fall 2024

Welcome to Chem 210! I am very excited to have you in this course. Please take some time to read and understand this syllabus. It outlines the resources you will engage with and course guidelines that I think will best help you learn about organic chemistry. I realize that it may look extensive, but do not feel overwhelmed – we will ensure that everything is easily accessible and provide ample reminders about upcoming deadlines. Above all, your TAs, LAs, and I are all here to help you have a positive experience and learn as much as possible. That means we are looking forward to your questions, so please don't hesitate to reach out!

Meeting Times: MWF 12:20 – 1:10 pm [redacted]
Th 10:35 – 11:50 am [redacted]

Course Instructor: Dr. Joseph Houck, [redacted]

Student Hours: See your course Canvas page for up-to-date times; you can always email us for an appointment!

Teaching Assistants: [redacted]

Learning Assistants: [redacted]

Required Materials:
- *Organic Chemistry*, by Brown, Iverson, Anslyn, & Foote, 8th Ed. with access to OWLv2 (a few options for purchase given on the syllabus page in Canvas.)
- Molecular model kit (cheapest from [redacted] due to bulk pricing; any kit will do but we prefer Molecular Visions Kit #3)
- Color copy of the ABCD voting card (on Canvas)

Recommended Materials:
- *Preparation for Organic Chemistry* by David Reingold (free ebook; link on Canvas)

Important Dates:
- August 31 at 11:59 pm is the deadline to adjust your schedule.
- November 15 at 11:59 pm is the last day to late drop the course with a "WN". [redacted] (Dates available at A complete course schedule can be found on Canvas.)

1 COURSE OBJECTIVES

During this course, students will learn concepts and principles of organic chemistry with reference to nomenclature of organic compounds, the relationship between structure and reactivity, organic spectroscopy, and the reactivities of saturated and unsaturated hydrocarbons (alkanes, alkenes, alkynes), as well as alkyl halides and alcohols. A second teaching goal is to share the importance of organic chemistry (with enthusiasm!) so that you may appreciate, and possibly even LIKE organic chemistry!

Learning Objectives. *After completing this course, you will be able to…*

- predict compound reactivity/function based on inspection of structure (identify electrophilic and nucleophilic sites within compounds).
- draw out and explain mechanisms of organic reactions for familiar and new addition, substitution, or elimination reactions.
- use spectroscopic data such from experiments such as mass spectrometry, IR spectroscopy, and NMR spectroscopy to determine the structure of organic compounds.
- make connections of organic chemistry to everyday life (biological processes, food chemistry, pharmaceuticals).
- characterize biomolecules and analyze biological reactions using principles of organic chemistry.

2 RESOURCES FOR SUCCESS

Few students have had much prior exposure to organic chemistry, so your biggest keys to success are an interest in learning new concepts and engaging with the many resources provided to you. The instructional team is committed to guiding you through this practice on your way to mastering the materials. Here are a few strategies and resources to support your success:

1. Please come to class with the required materials and be willing to actively participate. You are encouraged to complete the corresponding reading before coming to the class session and work the in-text problems. After class, you can then work through the homework problems. This approach will significantly improve your comprehension of the subject matter.
2. Ask questions! It is OK if you don't understand everything the first time. Take advantage of workshops, review sessions, and student hours. (Seriously, come see us!!!)
3. Review your class notes, compare them with the book, and make additional notes to clarify the subject matter.
4. **Work Problems!** I have prepared a list of appropriate end-of-chapter problems and topical problems sets that should be done (for the most part) WITHOUT your notes and text. Treat these problems as mini-exams and see if you can do the problems on your own. The biggest no-no is doing the problems with the answer in front of you. You don't learn by seeing the answer and saying 'oh, I can do that.' Prove to yourself that you can do it by simulating the testing environment as much as possible. *The more problems you tackle in this manner, the better you will do in the course.*
5. **Work in Groups!** The best way to learn something, or to find out what you don't really understand, is to try and teach it to someone else. (Trust me- I have learned more teaching than I did in the classroom as a student!) Leverage your network of peers!

6. Weekly Learning Community sessions are scheduled for CHEM 210. Teaching and learning assistants will facilitate workshops where you will solve relevant problems. These sessions are intended to be interactive and student driven.
7. You should anticipate spending between 6–9 hours each week studying (includes reading, doing homework, working problems, etc.) for this course and cannot afford to fall behind. For details on University expectations, please visit: [redacted]

3 CLASS SESSIONS

Research has shown that you can only learn a limited amount from lectures alone, no matter how clear or entertaining. People learn much more from being actively engaged and grappling with the material while learning it. Therefore, active participation and collaboration is an integral part of your learning in this course. Participation includes doing in-class activities and worksheets, answering in-class questions each day, and discussing your answers with peers in small groups. In addition to helping you learn, these participation activities provide vital feedback to me about how the class is going, and what everybody is understanding so I can adjust instruction accordingly in real time.

Items to bring to class *every day*:

- a pen or pencil
- note-taking material/class worksheet – we recommend a printed worksheet or digital version on a tablet, and
- voting card (described below)

Voting Cards: A multiple-choice voting card will be used each day. Please print a COLOR copy of this card (available for download on Canvas under course materials). If you forget yours one day, please ask the instructor before class for a spare "loaner" card for the day -- and please return it after class to ensure that there will always be loaners available for others. While your responses are not recorded or graded for correctness, it is to your (and my) great advantage to try to answer each question to the best of your ability. The responses provide both the students and the instructor with valuable feedback about what the class is understanding.

4 LEARNING TARGETS

- To **Complete** a Learning Target: Achieve *E or G* on a **Learning Target Quiz** for that target.

- To be **Proficient** on a Learning Target: Achieve *E or G* on a **Leaning Target Quiz** for a target that has already been completed.

Note: Some Learning Targets are considered Proficient as soon as they are Completed (1 time proficiency).

Target	Proficiency	Description
**Foundations***	1 time	Atomic structure, bonding, Lewis structure, functional groups, molecular structure
**LT1***	2 time	Convert between condensed structural formulas, Lewis structures, and bond-line structures, draw isomers
**LT2**	2 time	Mass spectrometry
**LT3**	2 time	IR spectroscopy
**LT4**	1 time	Organic Nomenclature
**LT5***	2 time	Identify and explain charge stability, formal charges, resonance, and aromaticity
**LT6**	2 time	Molecular Orbital Theory & Aromaticity
**LT7**	2 time	Conformation of Alkanes & Cylcoalkanes
**LT8***	2 time	Identify chiral and prochiral centers, indicate configurations, draw stereoisomers and identify relationships
**LT9***	2 time	Use structure or pK_a to explain acid-base properties of molecules and predict structure in various environments
**LT10***	2 time	Organic Reaction Types
**LT11***	2 time	Organic reaction mechanism basics: Nucs/Elecs & Mechanistic Steps
**LT12***	2 time	What if? Reactions and equilibria
**LT13**	2 time	S_N2 and E2 Reactions
**LT14**	2 time	S_N1 and E1 Reactions
**~~LT15~~**	~~2 time~~	Organic Synthesis – integrate into LT13, 14, 16, 17
**LT16**	2 time	Electrophilic alkene addition reactions
**LT17**	1 time	Radical stability, radical substitution of alkanes
**LT18**	1 time	NMR Signals, Splitting, Chemical Shift, Integration
**LT19**	1 time	MS, IR, NMR Structure Determination (take home assignment)
**LT20**[‡]	2 time	Chemistry Connections Case Studies
**Final Exam***	1 time	Final exam available during the scheduled final exam period.

*Denotes an essential target; necessary to achieve a grade of C or higher to be prepared for subsequent coursework
[‡]This learning target will be assessed through in-class activities

5 GRADING IN THE COURSE

Grading in this course is based on demonstrated mastery of concepts and skills. Learning is an inherently messy exercise. Students enter a course with different backgrounds. They learn at different rates. They make mistakes, a critical part of the learning process (you'll get to make lots of mistakes in this class), but even after they may a good understanding of the material. We understand this, and because want every student to master the material in this class by the end of the course, you will have numerous opportunities to demonstrate your mastery. Mistakes don't count against you in the class – if it takes you 4 tries to get something, but you get it, we consider you to be just as successful as someone who gets in on the first try! What we are interested in is not acquisition speed or required effort, but eventual understanding.

Your final grade is determined by the number of completed and proficient learning targets.

Grade	Complete LTs	Proficient LTs*	LTs with an *E* Grade
**F**	If the requirements for a D are not met		
**D**	8	0	0
**C**	9	9	0
**C+**	11	10	2
**B-**	13	11	4
**B**	15	13	6
**B+**	17	15	8
**A-**	19	17	10
**A**	21	19	12

*Proficient LTs must include the 9 essentials.

5.1 Learning Target Quizzes

Beginning in week 3, we will hold weekly quiz sessions on Thursdays where you take LTQs. You will typically take several short quizzes during a session with each quiz focusing on a single Learning Target.

- **Completing a Learning Targets**: You complete learning targets by earning a satisfactory grade on an LTQ.

- **Proficiency in a Learning Target**: You are proficient in a learning target by earning a satisfactory grade on a LTQ for a target you have previously completed.

  Some learning targets are labeled as "one-time proficiency". These targets are counted as both complete and proficient as soon as you earn one satisfactory grade on that target.

- Marks will be assigned to LT quizzes using an *EGRI* rubric.[1]

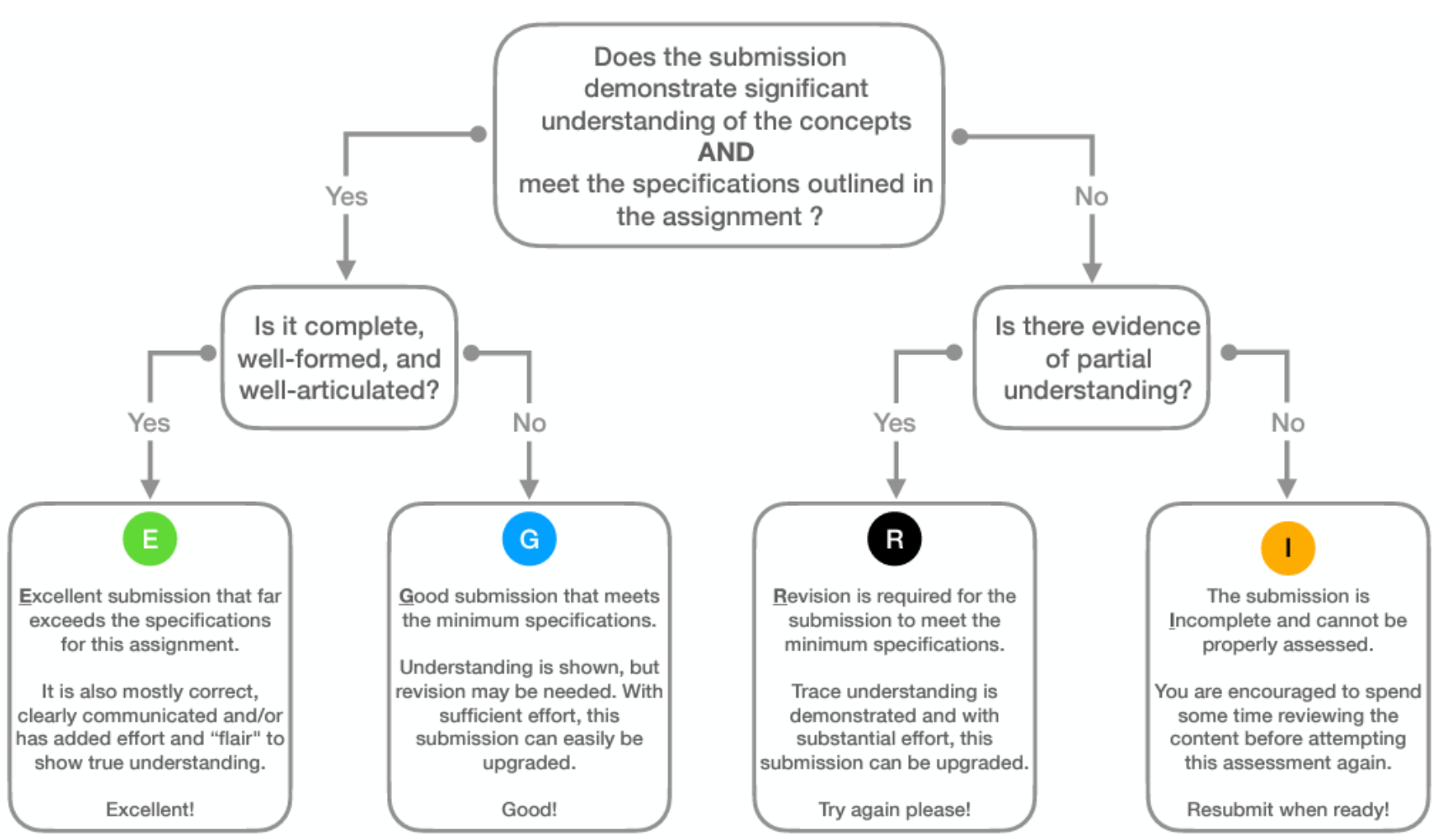

[1]Borrowed from Firas Moosvi and inspired by "EMRF: Everyday Rubric Grading", by Rodney Stutzman and Kimberly Race, and "Specifications grading with the EMRF rubric" by Robert Talbert.

- Participation in the Learning Target Quizzes each week is **critical**. Most learning targets will be available **four** times throughout the semester according to a schedule. Missing 1–2 quiz sessions throughout the semester is not likely to greatly impact your final grade. However, every time you miss a quiz session you miss an opportunity to complete or demonstrate proficiency in some targets and missing more than 1–2 sessions is likely to be detrimental.

- **Retaking a Learning Target Quiz**: If your LTQ is marked as needing revision or incomplete, you will have another opportunity to meet that target. After two unsatisfactory attempts on a Learning Target, you must meet with a member of the instructional team to review important concepts and must complete the associated assignments with the target. However, you need not wait before seeking assistance. Most learning targets will be available **four** times over the semester.

- **Important Note**: There are no points or percentage grades for Learning Target Quizzes. These assignments are either satisfactory or not.

5.2 Learning Target Quiz FAQ

Here are some anticipated questions about the learning target quizzes.

- What constitutes "*E or G*"?

  Each learning target quiz is an opportunity for you to demonstrate that you understand the concepts related to the Learning Target and that you have developed the skills associated with that target.

  A quiz will be graded *Excellent* if it far exceeds the specifications for this assignment. Think of this as A-level work (does not have to be perfect!). It is mostly correct, clearly communicated and/or has added effort and "flair" to show true understanding. A *Good* submission meets the minimum specifications (e.g. B-level work). Understanding is shown, but revision may be needed. With sufficient effort, this submission can easily be upgraded.

- Is "*revision*" on a Learning Target Quiz the same as an "F"?

  Not at all! We use "revision" to help emphasize that every time you attempt a learning target quiz, you have some understanding. Your final grade in the course is based, in part, on the number of Learning Targets you successfully complete and master. What matters is that you earn a "*E or G*" grade on each target. On some targets this may take multiple attempts, but each attempt is an opportunity for you to get feedback on your misunderstandings, to focus on what you don't yet understand about that particular target, and then to come back to that target with another attempt. This format will help you build understanding over time and strengthen your skills in every target in the course.

- Do I need to get a "*perfect score*" on a Learning Target to earn a "*satisfactory*"?

  Absolutely not! On any one learning target quiz, a "*E or G*" means that you have demonstrated proficiency and understanding in the particular skills included in that learning target. It may feel at first that this means you need to get a "100%" in everything, but this is a misconception. Both

"E or G" on a Learning Target Quiz is very different from getting a 100% on a one-time Midterm or Final Exam. Each Learning Target Quiz assesses a small set of skills focused around one Learning Target. After each attempt, you get feedback on that attempt. It is up to you to use this feedback to focus your review of the material before making another attempt. You earn a "*E or G"* on a Learning Target by building your understanding of that target to the point where you can demonstrate a strong understanding of that target in a short quiz. If you want to compare this process to a midterm exam, you can think of it as "getting 100%" on one small part of one exam, except that you have multiple attempts on the same part of that exam.

- Do I need to complete the Learning Targets in order?

  No. You do <u>not</u> need to get a "*E or G*" on LT1 before trying LT2 or LT3 (for example). Some learning targets will depend on skills developed on earlier targets and the material throughout the course is highly interconnected. It is important to continue attempting multiple learning target quizzes each week to keep pace with the course and to give yourself time for multiple attempts at each learning target. This approach differs from a setting with high-stakes exams. Each quiz attempt is simultaneously an assessment opportunity and a learning opportunity.

- How do I demonstrate proficiency in a Learning Target?

  Most Learning Targets are what we call **two-time proficiency**. This means you need to earn an *"E or G"* on that learning target twice during the semester, with some interval of time between completion and mastery (1 week minimum).

- How many times can I take a LTQ?

  You will have the opportunity to take most LTQs up to four (4) times. Learning targets will be available for two consecutive weeks in our Thursday sessions, followed by an additional two times during 'Quiz hours.'

- You've mentioned the possibility of taking LTQs during quiz hours. How does this work?

  As the semester progresses, some students may find they need to complete more quizzes than they can take in a single Thursday session. We will provide students an opportunity to sign up to take LTQs during quiz hours. Quiz hour opportunities will be reserved for targets where you have 2 or more attempts without a satisfactory grade. You are still limited to one attempt per learning target per week. A target may not be attempted more than one time in the same week.

5.3. Experience Points Modification

The number of learning targets completed or proficient may be modified by experience points (XP) earned during the course. Each modification earned will:

- increase the number of completed targets by one, **or**
- increase the number of proficient targets by one

Modifications are automatically applied at the end of the course in the way that will best benefit the student.

XP Earned	Effect
**Less than 360 XP**	No modifications
**360-470 XP**	1 modification
**471 or more XP**	2 modifications

You can earn experience points in the following ways (more details below). There are more than 600 XP available over the semester!

- Completing Foundations practice sets in Canvas (up to 5 XP per module)
- Completing daily questions and homework sets in OWLv2 (5-10 XP per assignment)
- Attending weekly Learning Communities with Learning Assistants (10 XP per week; sign up on Canvas in week 2)
- Completing practice sets in Canvas (XP varies per assignment)
- Completing other surveys and assignments as announced (several opportunities over the term)
- Having your name drawn from the hat for in-class participation (20 XP)

5.4 Earning Experience Points (XP)

Organic Foundations
The organic foundations module is an opportunity to practice and apply topics from general chemistry to the foundational concepts that are used to predict reactivity and physical properties of organic molecules. Throughout the semester, the foundational materials will be connected to topics covered in CHEM 210 so you will have multiple opportunities to build stronger connections to the driving forces of reactivity in organic chemistry. There is a series of videos to watch and five practice sets (Canvas quizzes) to complete in Canvas. You may take each 'quiz' as many times as you like. Only the highest score will be recorded. To demonstrate proficiency on the foundations topics, you will need to earn a 70% or higher on a 40-minute, multiple-choice quiz will be administered at Pollock Testing Center in week 2. You can take the quiz up to five times on the scheduled dates until you are proficient, earning a 70% or higher.

Daily Questions & HW
Homework is assigned to promote mastery of material. Mastery can only be achieved through practice and feedback. In addition, research on learning has shown that distributed practice (doing work over a period of time rather than doing all your weekly homework in one night) promotes better understanding and retention of material. Daily questions (each class day) and short homework sets (weekly) will be available to assist you in the practice, and you will receive feedback. We also want you to receive credit for your practice. To achieve the goals of practice, feedback and credit, please complete the assignments in OWLv2 (accessible in the Canvas modules) by each deadline in Canvas. There are a number of user resources such as guides and video tutorials available at: [redacted]

- Once you have registered and enrolled, you can log in at any time to complete or review your homework assignments. There are also additional exercises available for practice (not graded).

- During sign up – and throughout the term – if you have any technical problems or grading issues, please contact Cengage Support (via the headset icon in the upper right of the OWL page). The Cengage Learning support team is almost always faster and better able to resolve issues than your instructor.
- You have ten (10) attempts to answer each question.
- Each DQ will have a grace period of 24 hours.
- Each HW will be due have a grace period through Thursday with no penalty. Extensions beyond this time will not be granted for any reason. There are many ways to earn XP!

Learning Communities
Peer-led, team learning has been shown to have a positive impact on learning outcomes in a variety of STEM courses including Organic Chemistry. To facilitate this, there is a team of qualified Learning Assistants (LA) that will lead weekly sessions known as Learning Communities! The LAs are a wonderful resource who can share their experiences in the course. During weekly learning community meetings, you will get to know your leader and peers through check-ins, apply concepts in the course to biological topics, and have the opportunity to have your questions answered!

Names from a Hat
Additionally, you will have an opportunity to participate via "**Names from a Hat**." Periodically during class, I will draw individual names randomly from a hat to answer questions (typically after deliberation with your neighbors, so you are actually speaking for several people around you). Most names in the hat will get drawn at least once during the course of the semester, or possibly more. Responding when your name is called earns a point! All students will be given the option at the beginning of the semester of whether or not they would like their name to be included in the hat for the option of earning experience points this way. I request that only students who are planning to attend every class session (or very nearly so) put their names in the hat, so that I don't have to waste the class's time reading through names of students who are not present. If you wish to change your mind partway through the semester and have your name either added or removed from the hat, just ask.

5.4 Final Examination

A final exam will be scheduled during finals week, December 16-20. More details on the cumulative final exam will be provided when the date is scheduled. Please do not make end-of-semester travel plans until after the final exam schedule is published by the Registrar, typically in October.

6 COMMUNICATION

Course Management System
We will be using Canvas as the course management resource. If you are not familiar with Canvas, go to and log in using your directory ID (this is the same as your email username and password). After logging in, click on CHEM 210 to enter the site. You can monitor your grades and find course information here. I will also use the announcements feature to communicate with the class. Be sure to view these regularly

and adjust your email notifications accordingly. Note that comments on Canvas assignments do not get noticed; please reach out by email (described below).

Canvas Discussions
We will use Canvas discussions to facilitate informal communication with the class. A variety of boards will be available for various discussions between instructors & students as well as students & students. You can ask and answer questions here, which will be monitored by the TAs, LAs, and instructors. The best part of this forum is that the entire class can benefit from the questions and answers. More information on etiquette for using will be provided during the semester. Any personal inquiries can be addressed by email (see below).

Email (I strongly prefer an email rather than a Canvas message.)
I infrequently check my email after 8 PM and on weekends. If you email me during these times, I will most likely reply the following day or at the beginning of the week. If you do not hear from me within 48 hours (except weekends) you should probably follow up with another message.

While the University does not have rules and regulations for contacting faculty by email, I have a few rules of etiquette that students need to follow if they expect to receive a response: 1) there should be a salutation, e.g. Dear Dr. Houck 2) Please explain your situation using complete sentences. I do not text you and expect that you do not text me 3) Please give your **full name and course** at the end of the email. A sample acceptable email is shown below:

> *Dr. Houck,*
> *I need to miss class for a field trip next week. What advice do you have to stay caught up?*
> *John Smith*
> *CHEM 210, Section 3*

Several topics that will **NOT** be discussed via email:

1. Your grade in the course. You must schedule an appointment with me.
2. Missing grades on Canvas. (See above)
3. Issues and policies addressed in the syllabus. Check the syllabus first!
4. Other items previously announced via email or Canvas announcements.
5. Letters of recommendation. Please provide me with at least 1-month notice and come to see me in person/meet via Zoom. The more I get to know you, the better the letter will be!

7 POLICIES OF NOTE

Health & Wellness
(*policies mentioned here are subject to change based on updates from the University)
We realize that this Fall everyone may be experiencing a variety issues of reltated to health and wellness. We want you to know that we value your health and wellbeing. We are here for you and if you should need support, please feel free to reach out to your instructors anytime.

You will play an active role in your ability to stay healthy, so we ask that you take care of yourself. Please follow the University safety guidelines and recommendations. Do your best to maintain a balanced healthy lifestyle this semester by eating well, exercising, avoiding drugs and alcohol, getting enough sleep, and taking some time to relax. This will help you achieve your goals and cope with stress. Penn State hosts abundant opportunities for meaningful work and play. This abundance brings with it the challenge of maintaining a healthy, balanced life so you have will need to make thoughtful choices.

Masking
Our team recognizes that things are different now (post COVID-19 pandemic) than they were. You are welcome to wear a mask or a smile. Either is acceptable.

What if I need to miss a class session?
If you become ill or are exposed to someone who is sick, please do not come to class. We want you to know that you will be able to engage with the course material posted to Canvas after class. Assignment due dates and grading have been adjusted to accommodate absence with no penalty. The most important thing we ask is please keep connected with us. If you have difficulty with the course content, assignments, etc., due to illness, please reach out and we will try to work with you as best we can. We want you to learn and succeed. We want to have a wonderful experience learning with you. We are here for you.

Counseling and Psychological Services (CAPS)
Many students at Penn State face personal challenges or have psychological needs that may interfere with interfere with their academic progress, social development, or emotional wellbeing. The university offers a variety of confidential services to help you through difficult times, including individual and group counseling, crisis intervention, consultations, online chats, and mental health screenings. These services are provided by staff who welcome all students and embrace a philosophy respectful of clients' cultural and religious backgrounds, and sensitive to differences in race, ability, gender identity and sexual orientation.

[redacted]

Make-up quizzes
As required by University regulations, students who miss a quiz with a valid excuse (family emergency, illness, university-scheduled activities, etc.) will be given an opportunity to make it up. Prearranged make ups can be taken earlier in the quiz week. Alternatively, we can arrange a time to take the Learning Target Quiz as soon as you return.

1) The missed quiz is the result of a University-approved excuse. Per Senate Policy 42-27, "Requests for missing class or an evaluative event due to reasons that are based on false claims may be considered violations of the policy on Academic Integrity"

2) Your instructor must be notified **in writing** (email is preferred) **within 24 hours** of the missed quiz. You should include a written explanation of your absence within 1 week of the quiz date. This explanation need not include any unnecessary private details.

Deferred grades
Deferred grades are granted only in special circumstances when, for reasons beyond a student's control, a student is prevented from completing a course within the prescribed time. It is the student's obligation to request a deferred grade, and to take a comprehensive final exam before the University-set deadlines

Student Disability Resources (SDR)
Penn State welcomes students with disabilities into the University's educational programs. *If you have academic accommodations, please see me in my office for testing arrangements ASAP.* For further information, please visit Student Disability Resources Web site: [redacted] Their educational counselors can help with time management, reading, math learning skills, note-taking and exam preparation skills. All their services are *free* to PSU students.

In order to receive consideration for reasonable accommodations, you must contact SDR, participate in an intake interview, and provide documentation: [redacted] If the documentation supports your request for reasonable accommodations, your campus's disability services office will provide you with an accommodation letter. Please share this letter with your instructors and discuss the accommodations with them as early in your courses as possible. You must follow this process for every semester that you request accommodations.

Educational Equity/Report Bias
Penn State takes great pride to foster a diverse and inclusive environment for students, faculty, and staff. Acts of intolerance, discrimination, or harassment due to age, ancestry, color, disability, gender, gender identity, national origin, race, religious belief, sexual orientation, or veteran status are not tolerated and can be reported through Educational Equity via [redacted]

Honor Code Issues and Academic Integrity
You are reminded of the Academic Integrity Policy in the University Code of Conduct: "Academic integrity is the pursuit of scholarly activity in an open, honest and responsible manner. Academic integrity is a basic guiding principle for all academic activity at The Pennsylvania State University, and all members of the University community are expected to act in accordance with this principle. Consistent with this expectation, the University's Code of Conduct states that all students should act with personal integrity, respect other students' dignity, rights and property, and help create and maintain an environment in which all can succeed through the fruits of their efforts.

Academic integrity includes a commitment by all members of the University community *not to engage in or tolerate acts of falsification, misrepresentation or deception.* Such acts of dishonesty violate the fundamental ethical principles of the University community and compromise the worth of work completed by others."

For more information on Academic Integrity, please see the Undergraduate Advising Handbook: [redacted]

All Penn State [redacted] and Eberly College of Science [redacted] academic integrity policies and procedures regarding ethics and honorable behavior apply to this course. Academic dishonesty includes, but is not limited to, cheating, plagiarizing, fabrication of information or citations, facilitating acts of academic dishonesty by others, having unauthorized possession of examinations or other unauthorized class materials, submitting work of another person or work previously used without informing the instructor, or tampering with the academic work of other

students. In a broader context, you should be familiar with and follow the Penn State Code of Conduct and the Code of Mutual Respect and Cooperation.

Specific instances of academic dishonesty in this course include, but are not limited to:

- Representing yourself to be another person online
- Allowing another person to represent you online
- Allowing unauthorized persons to access lecture materials, quizzes, or exams
- Copying or helping someone else copy during an examination
- Receiving help or information from any person during a quiz or exam (this includes from websites such as Chegg and similar)
- Using unauthorized materials or notes during quizzes or examinations
- Searching for quiz or test answers on the internet ("googling" the answers)
- Using a text-programmable calculator on quizzes or examinations
- Using a cell phone or other communication device during a quiz or exam
- Stealing or destroying course materials
- Sharing information about quiz and exam questions with other students
- Altering answers or grades on graded examinations
- Having notes or extra papers of any kind out during an exam
- Having someone take an examination for you
- Keeping materials from a conflict or make-up exam
- Distributing course materials to others
- Providing a false excuse for missed exams
- Attempting to do any of the above

Consequences of such infractions may range from awarding a grade of “0” on the exam or assignment in question to receiving an immediate F in the course and not allowing the student to drop the class.

We are empathetic instructors. We will listen to you if you have a concern or something comes up. It is always better to be honest and talk to us than to resort to academic dishonesty. We thank you in advance for following these guidelines.
